# High Levels of Eomes Promote Exhaustion of Anti-tumor CD8^+^ T Cells

**DOI:** 10.3389/fimmu.2018.02981

**Published:** 2018-12-18

**Authors:** Jing Li, Yi He, Jing Hao, Ling Ni, Chen Dong

**Affiliations:** ^1^Institute for Immunology and School of Medicine, Tsinghua University, Beijing, China; ^2^Beijing Key Lab for Immunological Research on Chronic Diseases, Beijing, China

**Keywords:** eomes, transcription factor, T cell exhaustion, cytotoxic T cells, tumor immunity

## Abstract

Eomes, a T-box transcription factor, is known important for both function and homeostasis of effector and memory T cells, but was recently also implicated in CD8^+^ T cell exhaustion. However, whether and how Eomes might regulate effector functions or exhaustion of CD8^+^ T cells, especially in the tumor setting, is unknown. Here we first show, as tumor progressed, Eomes expression was elevated in tumor-infiltrating CD8^+^ T cells, especially in PD-1^+^Tim-3^+^ exhausted CD8^+^ T cells. Complete loss of Eomes in T cells resulted in impaired development of anti-tumor CTLs, whereas deletion of one allele of *Eomes* in T cells decreased development of exhausted CD8^+^ T cells, which offered better tumor control. Integrative analysis of RNAseq and ChIPseq of Eomes-overexpressing T cells revealed that high levels of Eomes expression directly controlled expression of T cell exhaustion genes, such as *Havcr2*. In addition, Eomes might compete with T-bet binding to regulatory genomic loci to antagonize T-bet functions. Collectively, Eomes exerts bimodal functions in CD8^+^ T cells in tumor.

## Introduction

When exposed to persistent antigens in chronic viral infection or cancer, effector CD8^+^ T cells acquire an alternative cell differentiation fate termed T cell exhaustion. They fail to undergo antigen-independent self-renewal like memory cells and lose their effector functions in a hierarchical manner, which hinders viral clearance and tumor control by these antigen-specific CD8^+^ T cells (1). Pathways implicated in regulating T cell exhaustion include persistent antigen exposure (2), co-expression of multiple inhibitory receptors (such as PD-1, Tim-3, and LAG3) (3) and cytokine signaling (IL-10, TGF-β, IFNα/β, and IL-27) (4–8). Exhausted CD8^+^ T cells have a transcriptional profile and epigenetic landscape markedly distinct from effector or memory CD8^+^ T cells (1, 9, 10), thus T cell exhaustion is now recognized as a specific and stable differentiation fate of CD8^+^ T cells.

Much effort has been made to identify the molecular mechanisms underlying CD8^+^ T cell exhaustion but so far it remains an unanswered question. Nonetheless, several transcription factors including Eomesodermin (Eomes), Blimp-1, von Hippel-Lindau tumor suppressor (VHL), Foxo1, IRF4, BATF, and NFATc1 have been implicated in CD8^+^ T cell exhaustion (11–15). Although these transcription factors also play some roles in other T cell lineages, they might have specific functions in exhausted CD8^+^ T cells.

Eomes, a paralogue of T-bet (encoded by *Tbx21*), is induced in effector CD8^+^ T cells and is required for T-bet-independent IFN-γ induction in CD8^+^ T cells. Ectopic expression of Eomes in Th2 cells is sufficient to invoke expression of cytotoxic T cell gene (e.g., perforin and granzyme B). Loss-of-function analyses suggest Eomes may complement with T-bet for full effector differentiation of CD8^+^ T cells (16). Later, it was reported that T-bet and Eomes are responsible for inducing CD122 expression and maintaining IL-15-dependent memory CD8^+^ T cells (17). But more recently, Eomes was found to be highly expressed in exhausted CD8^+^ T cells during chronic LCMV infection and Eomes^hi^PD-1^hi^ progeny co-expresses other inhibitory receptors and displays limited proliferative capacity (12). Eomes^hi^PD-1^hi^ population also positively correlates with severity of human HCV and HIV infection (12, 18). In addition, Eomes is directly involved in exhaustion of CD8^+^ TILs induced by B7S1 co-inhibitory pathway (19). However, it is not clear whether and how increased expression of Eomes promotes CD8^+^ T cell exhaustion.

In the current study, we analyzed the function of Eomes in CD8^+^ T cells in a cancer model. We found the amount of Eomes was increased in exhausted CD8^+^ T cells in the tumor. Eomes was haplosufficient for full effector differentiation of anti-tumor CTLs and high amounts of Eomes promoted exhausted phenotypes of CD8^+^ TILs. Furthermore, Eomes established the transcriptional profile associated with T cell exhaustion via binding to key regulatory loci on the genome. Overall, our data have offered important insights into the mechanism whereby Eomes mediates CD8^+^ T cell exhaustion.

## Materials and Methods

### Mice

C57BL/6J mice, *Cd4Cre* mice, *Eomes*^fl/fl^ mice (C57BL/6J background)(20) and OT-I mice were bred and kept under specific-pathogen free conditions in Animal Facility of Tsinghua University. *CD4Cre* was constructed by replacing the lck proximal promoter with the mCD4 promoter/enhancer/silencer (21). *Eomes*^fl/fl^ mice and *Eomes*^fl/fl^*Cd4Cre* mice were obtained by crossing *Eomes*^fl/fl^*Cd4Cre* mice and *Eomes*^fl/fl^ mice. *Eomes*^fl/+^ mice and *Eomes*^fl/+^*Cd4Cre* mice were obtained by crossing *Cd4Cre* mice and *Eomes*^fl/fl^ mice. All animal protocols are approved by governmental and institutional guidelines for animal welfare.

### Murine Tumor Models

E.G7 was purchased from ATCC. E.G7 was cultured in RPMI 1,640 medium with 2 mM L-glutamine adjusted to contain 10% FBS, 1.5 g/L sodium bicarbonate, 4.5 g/L glucose, 10 mM HEPES and 1.0 mM sodium pyruvate, 0.05 mM 2-mercaptoethanol and 0.4 mg/ml G418. 10^6^ E.G7 resuspended in 100 μL PBS was injected subcutaneously into 6~10-week-old C57BL/6J mice, *Eomes*^fl/fl^ mice, *Eomes*^fl/fl^*Cd4Cre* mice, *Eomes*^fl/+^ mice or *Eomes*^fl/+^*Cd4Cre* mice, and tumor growth was monitored every 3 days. Tumor volume was calculated by the following formula: tumor volume = 0.5 × length × width^2^.

### Isolation of TILs

E.G7 tumors were digested with 1 mg/mL collagenase D supplemented with 10 U/mL DNase I for 30 min at room temperature. Single cell suspension was centrifuged at a 40 and 70% discontinuous Percoll gradient (GE Healthcare) to isolate total tumor-infiltrating lymphocytes (TILs).

### Flow Cytometry

The following fluorescent dye-conjugated anti-mouse antibodies were used for staining: anti-CD8α (53-6.7), anti-PD-1 (J43), anti-Granzyme B (NGZB), anti-Perforin (ebio-omakd), anti-Foxp3 (FJK-16s), anti-IFN-γ (XMG1.2), anti-TOX (TXRX10) and anti-Eomes (Dan11mag) (eBioscience); anti-CD3e (145-2C11), anti-NK-1.1 (PK136), anti-CD4 (RM4-5), anti-CD44 (IM7), anti-CD62L (MEL-14), anti-IL-2 (JES6-5H4), anti-T-bet (O4-46) and anti-TNFα (MP6-XT22) (BD); anti-Tim-3 (RMT3-23) and anti-CD107a (1D4B) (Biolegend); anti-TCF1 (C63D9) (Cell Signaling Technology); BV421 labeled MHC tetramer H-2K^b^ SIINFEKL were obtained from NIH. Single cell suspensions were stained with antibodies against surface molecules. For tetramer staining, cells were incubated with BV421 labeled MHC tetramer H-2K^b^ SIINFEKL (1:2000, 4°C for 30 min) and washed twice prior to surface antibody staining. For intracellular cytokine staining, cells were stimulated with PMA (50 ng/mL, Sigma-Aldrich, MO) and ionomycin (500 ng/mL, Sigma-Aldrich, MO) in the presence of Brefeldin A (Golgiplug, BD Bioscience) for 4 h prior to staining with antibodies against surface proteins followed by fixation and permeabilization and staining with antibodies against intracellular antigens. Cells were analyzed on an LSRFortessa (BD) flow cytometer, and data analyzed using FlowJo X. Dead cells were excluded based on viability dye staining (Fixable viability dye eF506, eBioscience). Biexponential transformation was applied to display the flow cytometry data.

### *In vitro* Stimulation of CD8^+^ T Cells

CD8^+^ T cells were isolated from spleen and lymph nodes of *Eomes*^fl/fl^, *Eomes*^fl/fl^*Cd4Cre, Eomes*^fl/+^ or *Eomes*^fl/+^*Cd4Cre* mice using Dynabeads Flowcomp mouse CD8 kit (Invitrogen). For proliferation assay, CD8^+^ T cells were labeled with CFSE (2 μM CFSE, 37°C for 10 min) and cultured in 96-well plate coated with 1 μg/mL anti-CD3 or 1 μg/mL anti-CD3+1 μg/mL anti-CD28 (10^5^ per well) for 3 days. Proliferation capacity was evaluated by CFSE dilution using flow cytometry. To detect cytokine production, 10^5^ unlabeled CD8^+^ T cells were cultured n 96-well plate coated with 1 μg/mL anti-CD3 or 1μg/mL anti-CD3+1μg/mL anti-CD28 for 3 days. Golgi Plug was added 4 h prior to harvest and cytokine production were measured by intracellular flow cytometric analysis.

### Retroviral Overexpression of Eomes

Eomes was cloned into a retroviral expression vector (RVKM) which also encodes an IRES-hCD2 cassette. This vector was transfected into Pheonix to package retrovirus. The empty vector was used as a control. CD8^+^ T cells were isolated from spleen and lymph nodes of OT-I mice using Dynabeads Flowcomp mouse CD8 kit (Invitrogen). Then the cells were stimulated with SIINFEKL peptide (OVA257-264) at 2.5 ng/mL in the presence of 10 U/mL IL-2 for 24 hr. Retroviral supernatants were harvested, filtered, and supplemented with 6 μg/mL polybrene. OT-I T cell cultures were spinduced with the retroviral supernatant for 90 min at 1,800 rpm, 32°C. 48 h later, hCD2^+^ cells were sorted prior to re-stimulation or adoptive transfer. hCD2^+^ OT-I cells were plated at 4 × 10^4^ cells/well in 96-well plates and re-stimulated with 2.5 ng/mL OVA with 10 U/mL IL-2 for 3 days before harvested for RNAseq and ChIPseq analysis.

### Adoptive Transfer of Control or Eomes-Overexpressing OT-I Cells

1.5 × 10^6^ E.G7 was injected subcutaneously into 6~8-week-old female C57BL/6J mice. After 12 days, 0.5 × 10^6^ hCD2^+^ control or Eomes-overexpressing OT-I cells without re-stimulation was intravenously transferred into these mice. Tumor growth was monitored every 3 days.

### RNA Sequencing Analysis

Total RNA was extracted from re-stimulated control or Eomes-overexpressing OT-I cells and sent to BGI Genomics for library construction. The library products were sequenced via Illumina Hiseq4000 by BGI Genomics. The sequencing reads were filtered by SOAPnuke without quality problems. Genome mapping was done by HISAT. Clean reads were mapped to the mm10 reference genome using Bowtie2, and gene expression indicated by RPKM (Reads Per Kilobases per Million reads) was calculated by RSEM. Differentially expressed genes (DEG) were detected with PoissonDis by at least 1.5-fold change and FDR lower than 0.01. Gene set enrichment analysis (GSEA) was performed using (www.broadinstitute.org/gsea) (22). Pathway analysis of DEGs was conducted using DAVID Tools (https://david.ncifcrf.gov).

### ChIPseq Analysis

Re-stimulated control or Eomes-overexpressing OT-I cells were harvested, fixed with 1% formaldehyde for 10 min at room temperature and stopped by adding glycine to 125 mM. ChIP experiment was done following instructions of Active Motif's ChIP assay kit (53,035) with slight modifications, in which 10 cycles of sonication (Bioruptor) were added after enzyme digestion, and dynabeads protein G (Life Technologies) were used for immunoprecipitations. 5μg anti-TBR2/Eomes antibodies (ab23345, abcam) were used for each reaction. The precipitated DNA was sent to BGI genomics for library construction and deep sequencing. To analyze the sequencing data, the sequencing reads were mapped to the *Mus musculus* genome (version mm10) using Bowtie2 with no more than two mismatches. After PCR duplicates removal by samtools, MACS2 was used for peak calling, compared with each input control with FDR <0.001. Integrative analysis of RNAseq and ChIPseq data was conducted using BETA (23). mmTF motif analysis was computed using HOMER (homer.salk.edu).

### Statistical Analysis

Statistical analysis of tumor growth curves was performed using linear regression. Statistical comparisons were performed using ordinary One-way ANOVA analysis followed by multiple comparisons (more than two groups) or Student's *t*-test (comparing only two groups). *P*-values < 0.05 were considered to be statistically significant.

### Data and Software Availability

Gene Expression Omnibus: RNAseq data and ChIPseq data have been deposited under accession code GSE122895.

## Results

### Increased Expression of Eomes Is Associated With Exhausted CD8^+^ T Cells in Cancer

**Figure 1:**
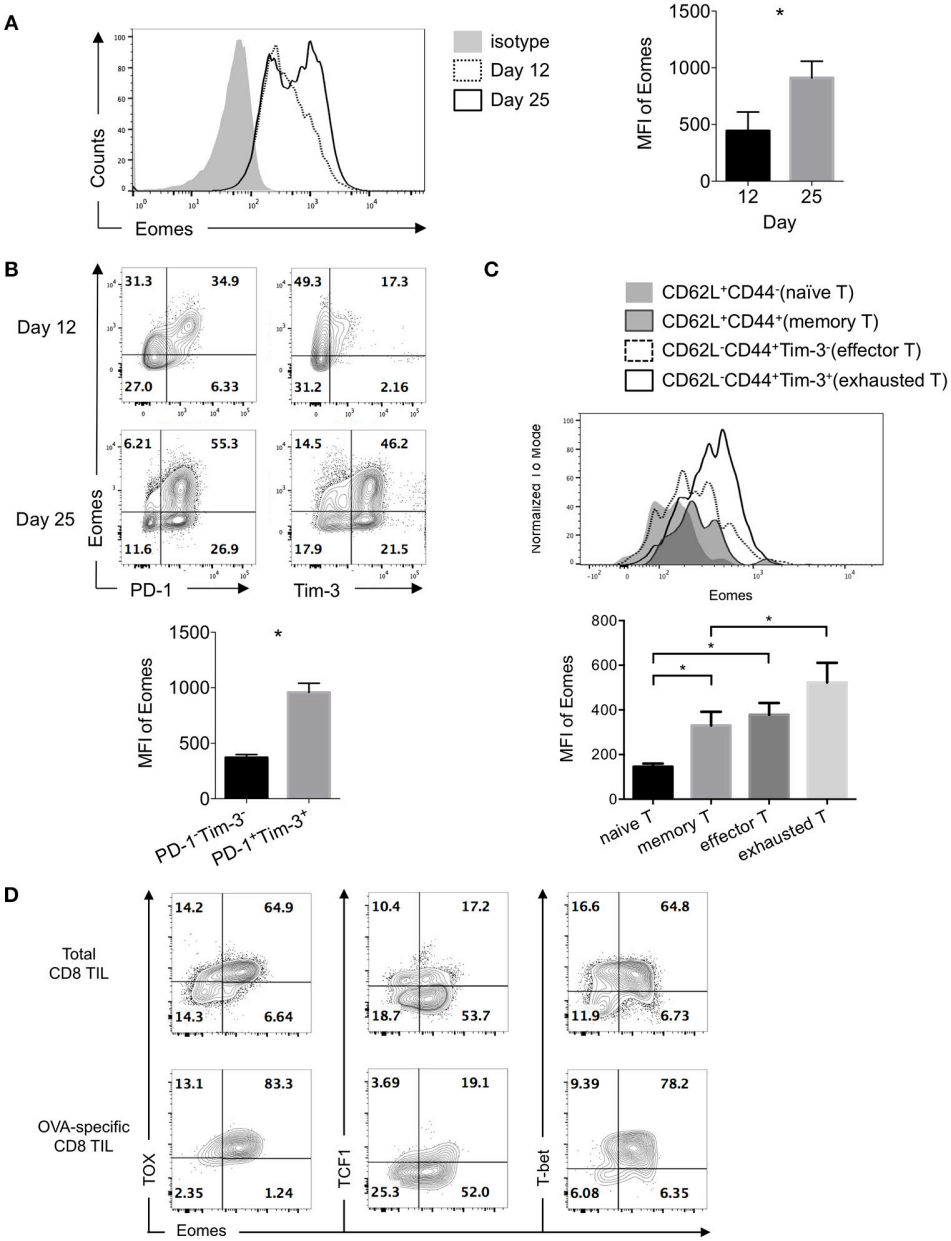
Exhausted CD8^+^ TILs express elevated Eomes. 10^6^ E.G7 cells were subcutaneously inoculated into C57BL/6 mice on Day 0. Tumor was harvested on Day 12 or Day 25 and TILs were isolated for flow cytometric analysis. **(A)** Representative (left) and summarized (right) histograms showing expression of Eomes in CD8^+^ TILs on Day 12 and Day 25 post tumor inoculation measured by intracellular flow cytometry. **(B)** Representative flow cytometric analysis of co-expression of Eomes with PD-1 or Tim-3 in CD8^+^ TILs on Day 12 and Day 25 (left) and summary histogram of expression of Eomes in PD-1^−^Tim-3^−^ and PD-1^+^Tim-3^+^ CD8^+^ TILs on Day 25 post tumor inoculation. **(C)** Expression of Eomes in CD8^+^ TILs at different differentiation stages. **(D)** Representative flow cytometric analysis of co-expression patterns of Eomes with TOX, TCF1 or T-bet in total CD8^+^ TILs and OVA-specific CD8^+^ TILs on Day 25 post tumor inoculation. Data are pooled from 2 independent experiments with 3–5 mice per group. Error bars denote mean ± SEM. Statistical analysis was performed using unpaired **(A)** and paired **(B)** Student's *t*-test and ordinary One-way ANOVA analysis followed by multiple comparisons **(C)** ^*^p < 0.05.

Expression of Eomesodermin (Eomes) was reported to be up-regulated in exhausted or terminally differentiated CD8^+^ T cells in chronic infection induced by LCMV clone 13 (12, 24). Previously we found B7S1 co-inhibitory pathway might drive T cell exhaustion through up-regulating Eomes (19). To assess Eomes expression in exhausted CD8^+^ T cells in the tumor, we examined CD8^+^ tumor-infiltrating T lymphocytes (TILs) at different time points after 10^6^ E.G7 cells were subcutaneously inoculated into C57BL/6 mice. Tumor was harvested on Day 12 or Day 25 and TILs were subject to flow cytometric analysis. Increased expression of Eomes was found in CD8^+^ TILs from Day 25 tumor compared to Day 12 (Figure 1A), indicating that Eomes expression in CD8^+^ TILs is elevated as tumor progresses. Moreover, Eomes was co-expressed with Programmed Death-1 (PD-1, encoded by *Pdcd1*) and T-cell immunoglobulin and mucin-domain containing-3 (Tim-3, encoded by *Havcr2*) in CD8^+^ TILs by day 12 to day 25 of tumor challenge. PD-1^+^Tim-3^+^ CD8^+^ TILs, which were previously known as exhausted T cells (25, 26), displayed higher expression of Eomes than PD-1^−^Tim-3^−^ cells (Figure 1B). We also compared the expression levels of Eomes in CD8^±^ TILs at different differentiation stages. Effector (CD62L^−^CD44^+^Tim-3^−^) and memory (CD62L^+^CD44^+^) CD8^±^ T cells in the tumor possessed higher Eomes expression than naïve T cells (CD62L^+^CD44^−^), but lower expression of Eomes than exhausted CD8^±^ TILs (CD62L^−^CD44^+^Tim-3^+^) (Figure 1C).

Thymocyte selection-associated HMG-box protein (TOX) was among the core exhaustion signatures identified by correlation matrix analysis of exhausted CD8^+^ T cells in HIV, together with PD-1, Eomes, 2B4, TIGIT and CD38 (27). Interestingly, we observed co-expression of Eomes with TOX in CD8^+^ TILs, especially in OVA-specific CD8^+^ TILs. However, much fewer Eomes-expressing cells co-expressed T cell factor 1 (TCF1, encoded by *Tcf7*), a transcription factor previously reported to repress exhaustion and maintain T cell stemness (28). In addition, T-bet and Eomes, the two homologous T-box transcription factors, were co-expressed in exhausted CD8^+^ TILs (Figure 1D). These findings suggest potential collaboration or competition of Eomes with other transcription factors.

### Eomes Is Required for Full Development of Anti-tumor CTLs

**Figure 2:**
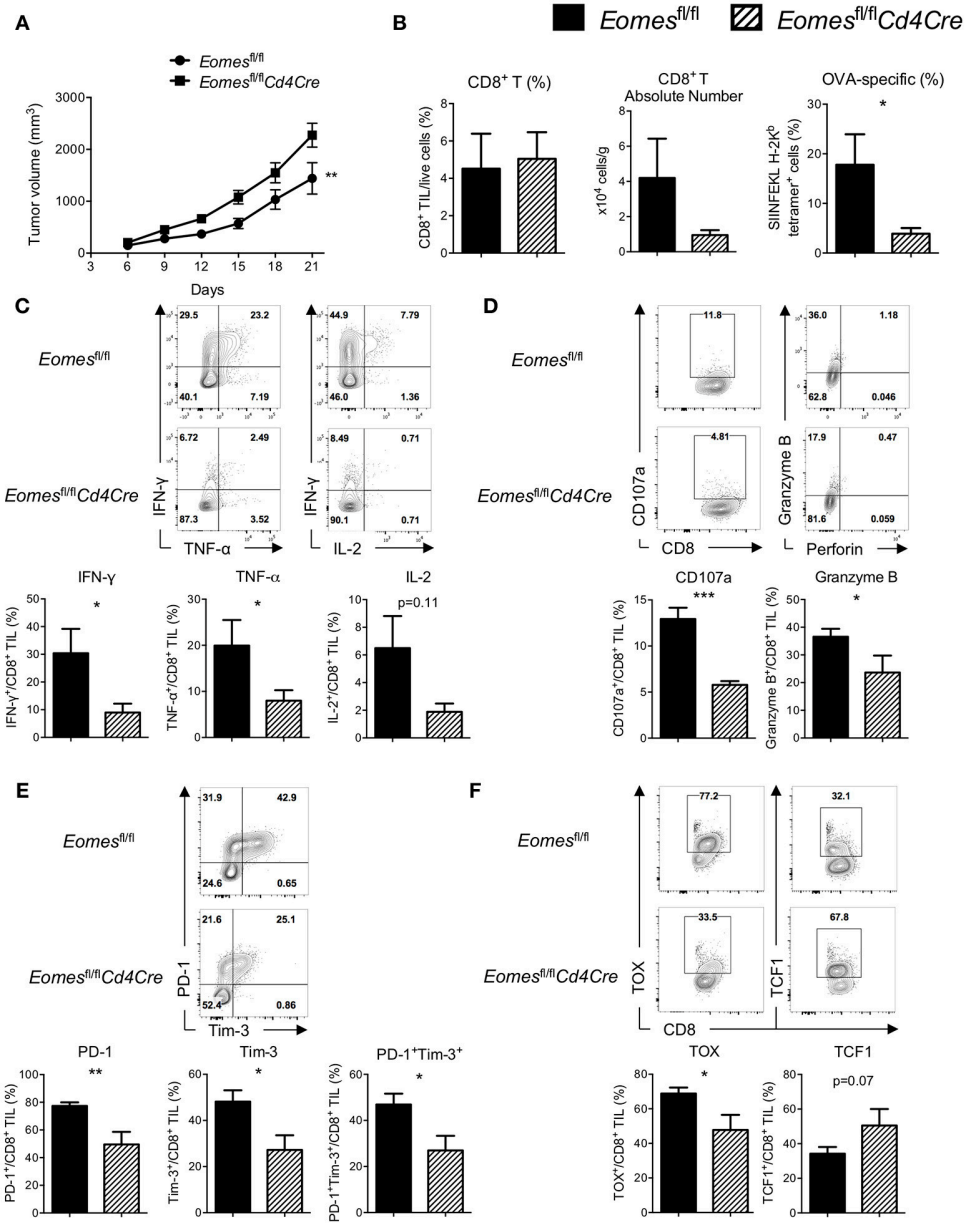
Eomes is required for full development of anti-tumor CTLs. **(A)** Mean tumor volume of intradermal E.G7 implants in *Eomes*^fl/fl^ vs. *Eomes*^fl/fl^*Cd4Cre* mice. **(B)** Summary histograms showing percentage of CD8^+^ TILs in total live cells, absolute number of CD8^+^ T cells in the tumor and frequency of OVA-specific CD8^+^ T (stained by SIINFEKL H-2K^b^ tetramer) in total CD8^+^ TILs from *Eomes*^fl/fl^ vs. *Eomes*^fl/fl^*Cd4Cre* mice on Day 21. **(C)** Production of IFN-γ, TNF-α and IL-2 by CD8^+^ TILs from *Eomes*^fl/fl^ vs. *Eomes*^fl/fl^*Cd4Cre* mice on Day 21 after PMA/ionomycin stimulation. **(D)** Baseline surface expression of CD107a and PMA/ionomycin-stimulated expression of intracellular Granzyme B in CD8^+^ TILs from *Eomes*^fl/fl^ vs. *Eomes*^fl/fl^*Cd4Cre* mice on Day 21. **(E)** Representative flow cytometric analysis and summary data of PD-1 and Tim-3 co-expression on CD8^+^ TILs from *Eomes*^fl/fl^ vs. *Eomes*^fl/fl^*Cd4Cre* mice on Day 21. **(F)** Expression of TOX and TCF1 in CD8^+^ TILs from *Eomes*^fl/fl^ vs. *Eomes*^fl/fl^*Cd4Cre* mice on Day 21 measured by intracellular flow cytometric analysis. Data are pooled from 2 independent experiments with 4–6 mice per group. Error bars denote mean ± SEM. Statistical analysis was performed using linear regression **(A)** or unpaired Student's *t*-test (others). ^*^p < 0.05, ^**^p < 0.01, ^***^p < 0.001.

Previous studies suggest that Eomes might complement with T-bet and act as a key transcription factor for full differentiation of effector CD8^+^ T cells (16). To evaluate the role of Eomes in development of anti-tumor CTLs, we challenged *Eomes*^fl/fl^ and *Eomes*^fl/fl^*Cd4Cre* (conditional ablation of *Eomes* in all T cells) mice with E.G7 tumor. Complete loss of Eomes in T cells resulted in accelerated tumor growth (Figure 2A) and reduced numbers of tumor antigen (OVA)-specific CD8^+^ T cells in the tumor (Figure 2B). Proportion of CD62L^−^CD44^+^ effector CD8^+^ T cells in spleen, tumor-draining lymph node (TDLN) and tumor was significantly decreased (Figures S1A,S1B). Deficiency of Eomes in T cells also dampened effector cytokine (IFN-γ and TNF-α) production and cytotoxicity (marked by surface CD107a expression and Granzyme B production) of CD8^+^ T cells in the tumor (Figures 2C,D), although effector functions of CD8^+^ T cells in spleen and TDLN was not affected (Figure S1C). *In vitro* stimulation of CD8^+^ T cells isolated from *Eomes*^fl/fl^ and *Eomes*^fl/fl^*Cd4Cre* mice by anti-CD3 or anti-CD3/CD28 also confirmed that complete loss of intrinsic Eomes led to impaired proliferation and effector functions of CD8^+^ T cells (Figures S2A,S2B). Moreover, expression of PD-1 and Tim-3, which are induced after T cell activation and then drive T cell exhaustion, was significantly decreased on CD8^+^ TILs from *Eomes*^fl/fl^*Cd4Cre* mice (Figure 2E). Complete absence of Eomes also increased TCF1 expression but decreased TOX expression in CD8^+^ TILs (Figure 2F), suggesting that loss of Eomes helps CD8^+^ T cells maintain cell stemness.

We then examined whether ablation of Eomes influences the numbers and effector function of CD4^+^ T cells in the tumor setting. Percentage of total CD4^+^ T cells (Figure S3A) and CD62L^−^CD44^+^ effector CD4^+^ T cells (Figure S3C) as well as their effector functions (Figure S3D) in spleen, TDLN and tumor were similar between *Eomes*^fl/fl^ and *Eomes*^fl/fl^*Cd4Cre* mice. Expression of PD-1 and Tim-3 on tumor-infiltrating CD4^+^ T cells was not altered by conditional knockout of Eomes in T cells, either (Figure S3E). However, frequency of regulatory T cells (Treg, Foxp3^+^CD4^+^ T) was reduced in the tumor of *Eomes*^fl/fl^*Cd4Cre* mice (Figure S3B), suggesting that Eomes might play a role in development or maintenance of intra-tumoral Treg cells instead of regulating effector functions of CD4^+^ T cells. Taken together, our data suggest that Eomes plays a key role in full development of anti-tumor CD8^+^ T cells.

### High Levels of Eomes Expression Is Required for T Cell Exhaustion

**Figure 3:**
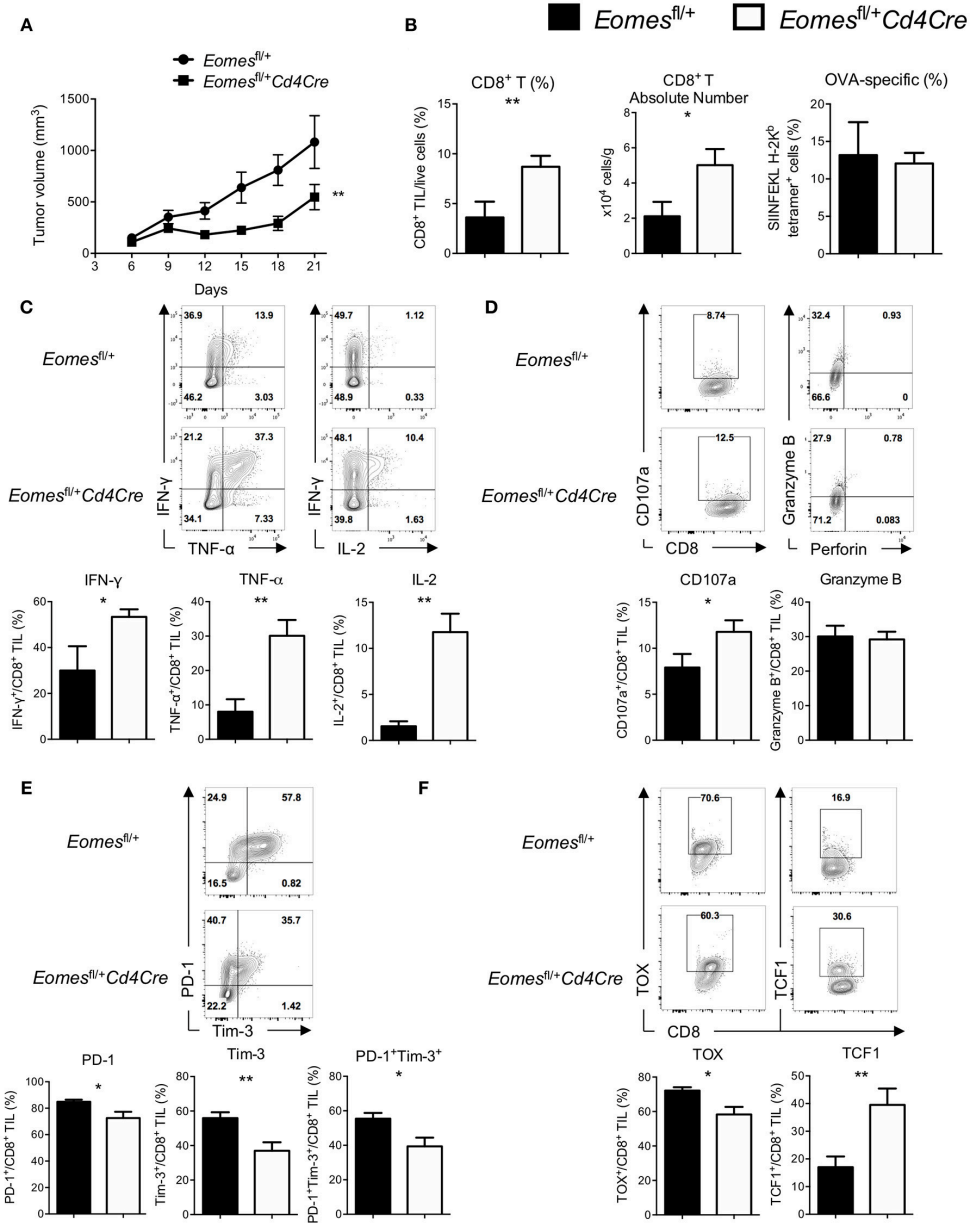
High amounts of Eomes in CD8^+^ TILs drive T cell exhaustion. **(A)** Mean tumor volume of intradermal E.G7 implants in *Eomes*^fl/+^ vs. *Eomes*^fl/+^*Cd4Cre* mice. **(B)** Summary histograms showing percentage of CD8^+^ TILs in total live cells, absolute number of CD8^+^ T cells in the tumor and frequency of OVA-specific CD8^+^ T (stained by SIINFEKL H-2K^b^ tetramer) in total CD8^+^ TILs from *Eomes*^fl/+^ vs. *Eomes*^fl/+^*Cd4Cre* mice on Day 21. **(C)** Production of IFN-γ, TNF-α and IL-2 by CD8^+^ TILs from *Eomes*^fl/+^ vs. *Eomes*^fl/+^*Cd4Cre* mice on Day 21 after PMA/ionomycin stimulation. **(D)** Baseline surface expression of CD107a and PMA/ionomycin-stimulated expression of intracellular Granzyme B in CD8^+^ TILs from *Eomes*^fl/+^ vs. *Eomes*^fl/+^*Cd4Cre* mice on Day 21. **(E)** Representative flow cytometric analysis and summary data of PD-1 and Tim-3 co-expression on CD8^+^ TILs from *Eomes*^fl/+^ vs. *Eomes*^fl/+^*Cd4Cre* mice on Day 21. **(F)** Expression of TOX and TCF1 in CD8^+^ TILs from *Eomes*^fl/+^ vs. *Eomes*^fl/+^*Cd4Cre* mice on Day 21 measured by intracellular flow cytometric analysis. Data are pooled from 2 independent experiments with 5–8 mice per group. Error bars denote mean ± SEM. Statistical analysis was performed using linear regression **(A)** or unpaired Student's *t*-test (others). ^*^p < 0.05, ^**^p < 0.01.

Although Eomes is required for full development of anti-tumor CTLs, increased expression of Eomes in CD8^+^ T cells is associated with T cell exhaustion. To examine the role of Eomes in T cell exhaustion, we generated mice carrying only one floxed *Eomes* allele on a *Cd4Cre* transgenic background (*Eomes*^fl/+^*Cd4Cre*) by crossing *Eomes*^fl/fl^ mice with *Cd4Cre*/+ mice, which might allow us to dissect the possible link between Eomes dose and T cell exhaustion while maintaining the basal dose of Eomes in CD8^+^ T cells for full CTL development. CD8^+^ T cells isolated from *Eomes*^fl/+^*Cd4Cre* mice did not have deficiency in proliferation or production of Granzyme B and IFN-γ upon anti-CD3 or anti-CD3/CD28 stimulation (Figures S4A,S4B), suggesting that Eomes is haplosufficient for CTL development. We then subcutaneously injected 10^6^ E.G7 tumor cells into *Eomes*^fl/+^*Cd4Cre* and *Eomes*^fl/+^ mice. Deletion of one allele of *Eomes* in T cells led to reduced tumor growth (Figure 3A) and increased frequencies and absolute numbers of CD8^+^ T cells in the tumor (Figure 3B). Percentages of CD62L^−^CD44^+^ effector CD8^+^ T cells were not significantly changed in spleen and TDLN (Figure S5B), but slightly increased in tumor of *Eomes*^fl/+^*Cd4Cre* mice. In addition, the amount of CD44 expression was significantly increased on CD8^+^ TILs from *Eomes*^fl/+^*Cd4Cre* mice (Figure S5A). CD8^+^ TILs from *Eomes*^fl/+^*Cd4Cre* mice showed enhanced production of IFN-γ, TNF-α, and IL-2 upon stimulation (Figure 3C) and increased CD107a surface expression (Figure 3D) compared to CD8^+^ TILs from *Eomes*^fl/+^ mice. TNF-α production by CD8^+^ T cells from TDLN was also increased in *Eomes*^fl/+^*Cd4Cre* mice (Figure S5C). Furthermore, deficiency of one allele of *Eomes* in T cells resulted in decreased co-expression of PD-1 and Tim-3, which marks T cell exhaustion, on tumor-infiltrating CD8^+^ T cells (Figure 3E). Exhaustion-associated transcription factor TOX was decreased, while TCF1, a transcription factor associated with T cell stemness and memory, was increased in CD8^+^ TILs from *Eomes*^fl/+^*Cd4Cre* mice (Figure 3F). These results indicate that CD8^+^ TILs in *Eomes*^fl/+^*Cd4Cre* mice were less exhausted. However, conditional deletion of one *Eomes* allele in T cells did not alter the number or phenotypes of CD4^+^ T cells in E.G7 tumor model (Figure S6).

To further investigate the dosage effects of Eomes on T cell function, we overexpressed Eomes in *in vitro* activated OT-I cells and then adoptively transferred them into E.G7-bearing mice to compare their *in vivo* cytolytic activity with control OT-I cells. Overexpression of Eomes in OT-I cells dampened their capability to control tumor growth *in vivo* (Figure S7). Therefore, high levels of Eomes expression in tumor-infiltrating CD8^+^ T cells are required for their exhausted phenotypes.

### Eomes Controls the Transcriptional Profile Associated With Exhaustion

**Figure 4:**
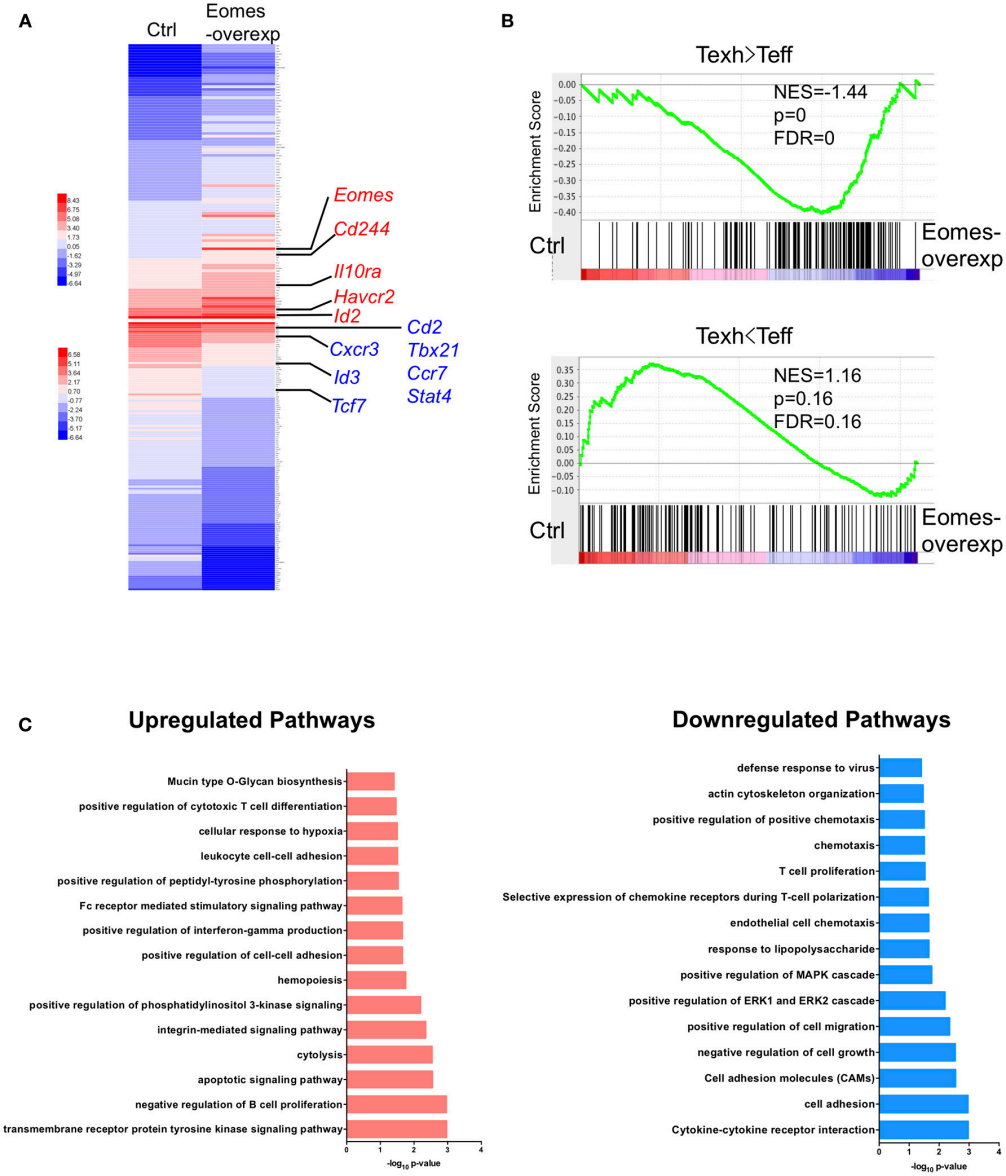
Eomes establishes the exhaustion-associated transcriptional profile. **(A)** Heatmap of the top 100 up-regulated and down-regulated differentially expressed genes (DEGs) in Eomes-overexpressing vs. control OT-I cells. Genes important for Th1 differentiation and memory development as well as for T cell exhaustion are highlighted. See also Table S1. **(B)** Gene set enrichment analysis (GSEA) of genes up-regulated and down-regulated in exhausted CD8^+^ T cells (Day 30) compared to effector CD8^+^ T cells (Day 15) in chronic LCMV infection (30). **(C)** Pathway analysis of up-regulated and down-regulated DEGs using DAVID.

To understand how high levels of Eomes drive T cell exhaustion, we conducted trancriptomic profiling on OT-I cells chronically stimulated with OVA peptide (SIINFEKL) *in vitro* with or without Eomes overexpression. Previously we showed that overexpression of Eomes decreased cytokine production by OT-I cells in this system (19). Two hundred and forty-two genes were found up-regulated and 513 genes down-regulated by Eomes overexpression (Table S1, FDR <0.01, fold change>1.5). Eomes-overexpressing OT-I cells showed increased transcript abundance of genes encoding co-inhibitory receptors (e.g., *Cd244* and *Havcr2*) and *Il10ra* which encodes receptor for IL-10, a cytokine that promotes T cell exhaustion (29). In contrast, Eomes-overexpressing OT-I cells down-regulated genes associated with memory development, including *Ccr7* and *Tcf7*, and genes important for Th1 differentiation, e.g., *Tbx21, Stat4*, and *Cxcr3* (Figure 4A). Gene set enrichment analysis (GSEA) revealed that genes up-regulated in exhausted CD8^+^ T cells during chronic viral infection were enriched, while effector signatures were reduced in Eomes-overexpressing OT-I compared to control OT-I cells (Figure 4B). Pathway analysis revealed up-regulation of CTL differentiation and cytolysis pathways by Eomes overexpression, validating the important role of Eomes in CTL development. However, Eomes overexpression also induced up-regulation of genes involved in apoptosis and hypoxia pathways, while down-regulating T cell proliferation, chemotaxis and cytokine pathways (Figure 4C), suggesting that Eomes also negatively regulates T cell function. Collectively, our findings demonstrate that high amounts of Eomes control the transcriptional profile associated with T cell exhaustion.

### Eomes Binds to Signature Genes of Exhausted T Cells

**Figure 5:**
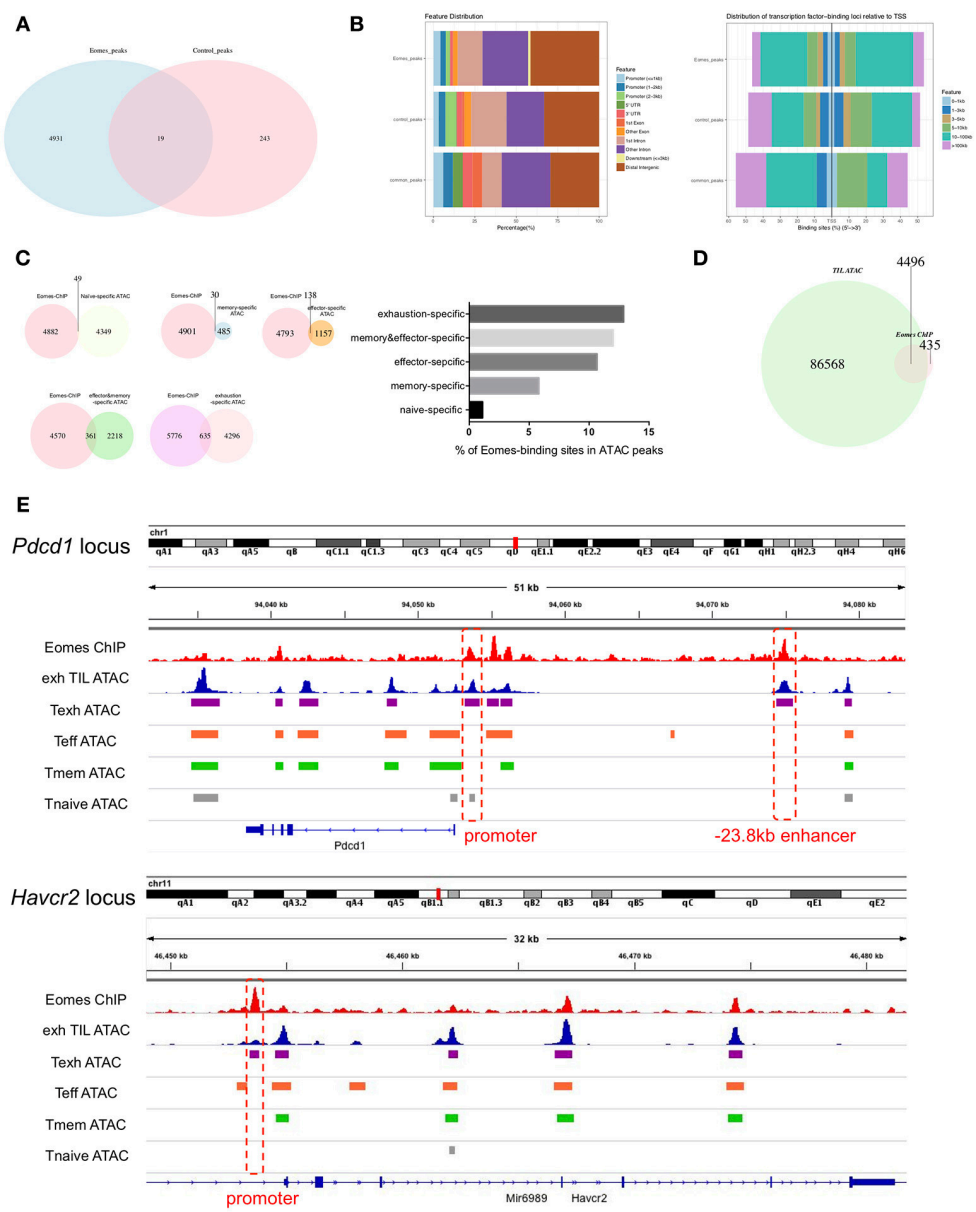
Eomes binds to key genomic loci to establish the transcriptional profile of T cell exhaustion. **(A)** Venn diagram of unique and common peaks identified from Eomes-ChIPseq of control vs. Eomes-overexpressing OT-I cells. **(B)** Genomic feature distribution and distribution of Eomes-binding loci relative to TSS of peaks indicated in **(A). (C)** Venn diagrams showing overlapping peaks between Eomes-binding sites identified by Eomes-ChIPseq and naïve, memory, effector or exhaustion-specific open chromatin regions (OCRs) identified by ATACseq (9), and summary histogram showing percentage of Eomes-binding sites in naïve, memory, effector or exhaustion-specific OCRs. **(D)** Venn diagram showing overlapping peaks between Eomes-binding sites and OCRs of exhausted CD8^+^ T cell in the tumor identified by ATACseq (19). **(E)** Representative Eomes-ChIPseq tracks aligned with previously reported ATACseq tracks of exhausted CD8^+^ TILs (19) and naïve, effector, memory or exhausted CD8^+^ T cells in chronic LCMV infection (9) at the *Pdcd1* and *Havcr2* locus. Promoter and enhancer regions are highlighted.

To investigate how Eomes functions as a transcription factor to mediate transcriptional profiles of T cell exhaustion, we performed chromatin immunoprecipitation coupled with high-throughput sequencing (ChIPseq) on *in vitro* cultured control vs. Eomes-overexpressing OT-I cells using anti-Eomes antibodies to identify the genome-wide binding sites of Eomes. Four thousand, nine hundred thirty-one peaks were identified specifically in Eomes-overexpressing OT-I cells (Figure 5A), while only 243 peaks were specific to control OT-I cells likely due to low Eomes expression (Figure 5A). Therefore, we focused on these 4,931 peaks for further analysis. Analyses of genomic feature distribution of the 4,931 peaks showed that most of the Eomes-binding events fell into introns or intergenic regions, mostly 10–100 kb upstream or downstream of transcription start sites (TSS), indicating that Eomes often binds to enhancers or distal regulatory elements to regulate gene transcription (Figure 5B). We compared the Eomes-binding sites with the naïve, memory, effector or exhaustion-specific open chromatin regions (OCRs) identified by ATAC-seq analyses of naïve, memory, effector or exhausted CD8^+^ T cells during chronic LCMV infection (9). There were more Eomes-binding events falling into effector-specific and exhaustion-specific OCRs (Figure 5C), suggesting that Eomes mainly functions in effector and exhausted cells. We also detected the overlapping peaks between Eomes-ChIPseq and ATAC-seq of exhausted CD8^+^ TILs (19). Over 90% of Eomes-binding sites were within the OCRs of exhausted CD8^+^ TILs, which indicates that Eomes binds to the open genomic loci to control transcriptional profile of T cell exhaustion in tumor (Figure 5D). Therefore, we aligned the Eomes-ChIPseq tracks with ATAC-seq tracks of exhausted CD8^+^ TIL and naive, effector, memory or exhausted CD8^+^ T cells in chronic viral infection to see whether Eomes binds to key genomic loci that regulates transcription of exhaustion-associated genes. We found that Eomes bound to the promoter and −23.8 kb enhancer of *Pdcd1*, which were accessible only in exhausted CD8^+^ T cells in tumor or during chronic viral infection (Figure 5E). Previous studies *d*emonstrated that the−23.8 kb enhancer of *Pdcd1* is required to maintain consistently high levels of PD-1 expression in exhausted CD8^+^ T cells (10). Accordingly, deletion of one or two copies of *Eomes* in T cells resulted in significant decrease of PD-1 expression on CD8^+^ TILs (Figures 2E, 3E), indicating that Eomes maintains high PD-1 expression on exhausted CD8^+^ T cells through binding to key genomic regulatory elements of *Pdcd1*. Eomes also bound to the promoter region of *Havcr2* locus, which became accessible only in exhausted CD8^+^ T cells (Figure 5E). Consistent with this finding, Tim-3, encoded by the *Havcr2* gene, was down-regulated by ablation of one or two alleles of *Eomes* (Figures 2E, 3E) and up-regulated by Eomes overexpression (Figure 4A) in CD8^+^ T cells, suggesting that Eomes might promote expression of Tim-3 on exhausted CD8^+^ T cells by acting on its promoter.

**Figure 6:**
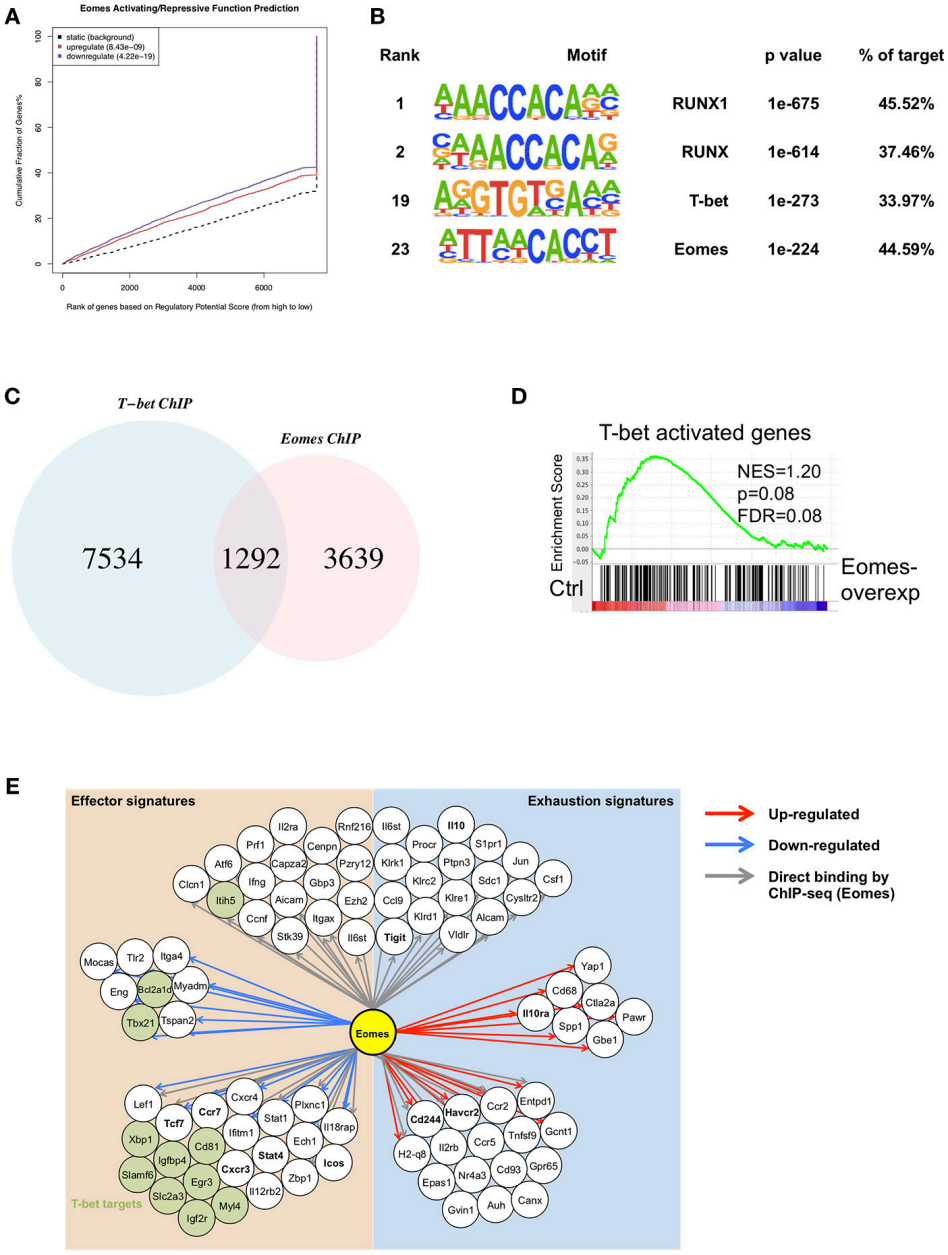
Integrative analysis of ChIPseq and RNAseq data. **(A)** BETA analysis predicting activating/repressive function of Eomes in CD8^+^ T cells. *p*-values represent the significance of the up or down group calculated by the Kolmogorov-Smirnov test. **(B)** Transcription factor motif enrichment in ChIPseq peaks using HOMER. **(C)** Venn diagram showing overlapping peaks between Eomes-binding sites and T-bet-binding sites identified by ChIPseq. **(D)** GSEA of T-bet-activated genes. Genes from the left to right of the rank-ordered list are enriched in control and Eomes-overexpressing OT-I cells, respectively. **(E)** Network model based on RNAseq data of Eomes-overexpressing vs. control OT-I cells and Eomes binding events (ChIPseq data for Eomes). Red arrows designate genes up-regulated by Eomes, blue arrows designate genes down-regulated by Eomes, and gray arrows denote Eomes-binding events on associated genomic loci.

In order to identify global gene targets of Eomes, we performed BETA analysis to integrate Eomes binding with gene expression altered by Eomes overexpression (23, 31). Eomes was predicted to possess both activating and repressive functions (Figure 6A). This analysis also predicted 2,625 direct regulatory targets of Eomes (1,195 up-regulated and 1,430 down-regulated, Table S2). To explore possible interaction or regulation to other transcription factors by Eomes, we conducted transcription factor motif analysis on Eomes-binding sites. Eomes-binding sites identified by ChIP-seq of Eomes-overexpressing OT-I cells showed enrichment for T-bet binding motifs (Figure 6B and Table S3). Consistently, we detected 26% of Eomes-binding sites overlapped with T-bet-binding sites when comparing our Eomes-ChIPseq data with T-bet-ChIPseq (32) (Figure 6C). Moreover, GSEA revealed that T-bet activated were down-regulated in Eomes-overexpressing OT-I cells (Figure 6D), indicating Eomes might compete with T-bet binding to regulatory genomic loci to repress transcription of its target genes. Therefore, we summarized a network model based on transcriptional profile altered by Eomes overexpression and Eomes binding events on associated genomic loci (Figure 6E). This model showed that Eomes could up-regulate exhaustion-associated genes and down-regulate effector genes by its direct binding to their regulatory genomic loci or by indirect regulation, thus conferring exhaustion fate to chronically stimulated T cells.

## Discussion

The molecular mechanism underlying CD8^+^ T cell exhaustion has not been well understood. Here we show up-regulated expression of Eomes in exhausted CD8^+^ T cells in the tumor. Eomes was required for full development of anti-tumor CTLs, but high amounts of Eomes in CD8^+^ TILs drove T cell exhaustion and suppressed their effector functions. Moreover, Eomes can bind to key genomic loci to control transcriptional signatures for T cell exhaustion.

Association of Eomes with CD8^+^ T cell exhaustion was first implicated by the findings of high levels of Eomes expression in exhausted CD8^+^ T cells during chronic LCMV infection (12). Eomes^hi^PD-1^hi^ population also positively correlated with severity of human HCV and HIV infection (12, 18). Recently, it was reported that increased Eomes expression was found in CD8^+^ T cells with an exhausted phenotype in mice with relapsed multiple myeloma (33). In our study, we found that Eomes expression was elevated in tumor-infiltrating CD8^+^ T cells as tumor progressed, and exhausted CD8^+^ TILs (characterized by co-expression of PD-1 and Tim-3) possessed enhanced expression of Eomes than naïve, memory and effector cells in tumor. In the context of human cancer, it was previously reported that PD-1^+^ Tim-3^+^ CD8^+^ TILs expressed higher EOMES transcripts than PD-1^−^Tim-3^−^ CD8^+^ TILs in head and neck cancer patients (34). Moreover, in myeloma patients, tumor load above 10% plasma cells was associated with increased number of PD-1^+^EOMES^high^T-bet^low^ CD8^+^ T cells (35). Therefore, EOMES might also play an important role in regulating T cell exhaustion in human tumors.

Eomes seems to play complex roles in effector and exhaustion differentiation of CD8^+^ T cells: on one hand, it cooperates with T-bet for full effector differentiation (16); on the other hand, it is highly associated with T cell exhaustion and terminal differentiation (12). Based on our findings, these context-dependent activities of Eomes may be due to different expression levels of Eomes in effector and exhausted CD8^+^ T cells. Basal dosage of Eomes is required for initial expansion and effector development of CD8^+^ T cells, since complete deletion of Eomes in T cells led to impaired effector differentiation of anti-tumor CTLs. Deletion of one allele of *Eomes* in CD8^+^ T cells resulted in less severe exhausted phenotypes, while overexpression of Eomes in OT-I cells dampened their *in vivo* cytotoxicity, indicating that high amounts of Eomes drive T cell exhaustion instead of promoting effector functions of CD8^+^ T cells. The dosage or concentration of Eomes in cells might affect its binding to different genomic sites: at low concentration it might only bind to high affinity sites, while at high concentration its binding might occur more broadly (binding to both high and low affinity sites). It is also possible that the distinct epigenetic landscapes of effector and exhausted CD8^+^ T cells alter the accessibility of Eomes to its binding sites on the genome, as comparison of Eomes-ChIPseq and ATACseq data revealed that Eomes can bind to chromatin regions specifically open in effector or exhausted CD8^+^ T cells. Another possibility is that Eomes might have different binding partners in effector and exhausted CD8^+^ T cells, which needs to be further investigated by co-IP experiments.

In chronic viral infection, T-bet and Eomes cooperate to sustain the exhausted CD8^+^ T cell pool: the T-bet^hi^PD-1^mid^ progenitor pool retains some proliferative potential, while the Eomes^hi^PD-1^hi^ terminal progeny has limited proliferative capacity and weaker cytokine production; the T-bet^hi^PD-1^mid^ subpopulation can lose T-bet expression and convert to the Eomes^hi^PD-1^hi^ terminal progeny, indicating exclusive expression and opposite functions of T-bet and Eomes in exhausted CD8^+^ T cells (12). However, in the tumor setting, Eomes tends to be co-expressed with T-bet in exhausted CD8^+^ T cells, although overexpression of Eomes down-regulated *Tbx21* transcripts that encode T-bet. We found 26% of Eomes-binding sites overlapped with T-bet-binding sites and T-bet-activated target genes were down-regulated by Eomes overexpression, suggesting that high amounts of Eomes in exhausted CD8^+^ T cells might compete with T-bet for its genomic binding sites to antagonize T-bet functions. However, precise biochemical studies and ChIP analyses are needed to further confirm this regulatory mechanism.

Transcription factors can exert activating or repressive functions to regulate target gene expression. Our BETA analysis demonstrates that Eomes possessed both activating and repressive functions. It can directly bind to regulatory genomic sites as an activating or repressive transcription factor to up-regulate transcription of exhaustion-associated genes (e.g., *Cd244* and *Havcr2*) or down-regulate transcription of effector/memory-associated genes (e.g., *Cxcr3, Stat4, Tcf7*, and *Ccr7*). It can also control gene expression by indirect regulation (e.g., up-regulated: *Il10ra*; down-regulated: *Tbx21*). Overall, our findings reveal that high amounts of Eomes establish transcriptional profile of exhausted CD8^+^ T cells, mainly through its activating and repressive transcription factor activities. Further studies are required to determine the regulatory networks of Eomes in effector and exhausted CD8^+^ T cells and how to modulate the dose and function of Eomes in CD8^+^ T cells to reverse T cell exhaustion.

## Ethics Statement

All the animal experiments were performed with the use of protocols approved by the Institutional Animal Care and Use Committee of Tsinghua University.

## Author Contributions

JL performed most of the experiments. YH performed analysis of ChIPseq data. JH and LN performed some of the experiments or contributed to their design. JL and CD wrote the manuscript. CD supervised the study.

### Conflict of Interest Statement

The authors declare that the research was conducted in the absence of any commercial or financial relationships that could be construed as a potential conflict of interest.

## References

[1] Wherry EJ. T cell exhaustion. Nat Immunol. (2011) 12:492–9. 10.1038/ni.2035 21739672

[2] Angelosanto JM Blackburn SD Crawford A Wherry EJ. Progressive loss of memory T cell potential and commitment to exhaustion during chronic viral infection. J Virol. (2012) 86:8161–70. 10.1128/JVI.00889-12 22623779 PMC3421680

[3] Blackburn SD Shin H Haining WN Zou T Workman CJ Polley A. Coregulation of CD8+ T cell exhaustion by multiple inhibitory receptors during chronic viral infection. Nat Immunol. (2009) 10:29–37. 10.1038/ni.1679 19043418 PMC2605166

[4] Wherry EJ Kurachi M. Molecular and cellular insights into T cell exhaustion. Nat Rev Immunol. (2015) 15:486–99. 10.1038/nri3862 26205583 PMC4889009

[5] Brooks DG Trifilo MJ Edelmann KH Teyton L McGavern DB Oldstone MB Interleukin-10 determines viral clearance or persistence *in vivo*. Nat Med. (2006) 12:1301–9. 10.1038/nm1492 17041596 PMC2535582

[6] Tinoco R Alcalde V Yang Y Sauer K Zuniga EI. Cell-intrinsic transforming growth factor-beta signaling mediates virus-specific CD8+ T cell deletion and viral persistence *in vivo*. Immunity (2009) 31:145–57. 10.1016/j.immuni.2009.06.015 19604493 PMC3039716

[7] Teijaro JR Ng C Lee AM Sullivan BM Sheehan KCF Welch M. Persistent LCMV infection is controlled by blockade of type I interferon signaling. Science (2013) 340:207–11. 10.1126/science.1235214 23580529 PMC3640797

[8] Chihara N Madi A Kondo T Zhang H Acharya N Singer M. Induction and transcriptional regulation of the co-inhibitory gene module in T cells. Nature (2018) 558:454–9. 10.1038/s41586-018-0206-z 29899446 PMC6130914

[9] Pauken KE Sammons MA Odorizzi PM Manne S Godec J Khan O. Epigenetic stability of exhausted T cells limits durability of reinvigoration by PD-1 blockade. Science (2016) 354:1160–5. 10.1126/science.aaf2807 27789795 PMC5484795

[10] Sen DR Kaminski J Barnitz RA Kurachi M Gerdemann U Yates KB. The epigenetic landscape of T cell exhaustion. Science (2016) 354:1165–9. 10.1126/science.aae0491 27789799 PMC5497589

[11] Shin H Blackburn SD Intlekofer AM Kao C Angelosanto JM Reiner SL. A role for the transcriptional repressor Blimp-1 in CD8(+) T cell exhaustion during chronic viral infection. Immunity (2009) 31:309–20. 10.1016/j.immuni.2009.06.019 19664943 PMC2747257

[12] Paley MA Kroy DC Odorizzi PM Johnnidis JB Dolfi DV Barnett BE. Progenitor and terminal subsets of CD8+ T cells cooperate to contain chronic viral infection. Science (2012) 338:1220–5. 10.1126/science.1229620 23197535 PMC3653769

[13] Doedens AL Phan AT Stradner MH Fujimoto JK Nguyen JV Yang E. Hypoxia-inducible factors enhance the effector responses of CD8(+) T cells to persistent antigen. Nat Immunol. (2013) 14:1173–82. 10.1038/ni.2714 24076634 PMC3977965

[14] Staron MM Gray SM Marshall HD Parish IA Chen JH Perry CJ. The transcription factor FoxO1 sustains expression of the inhibitory receptor PD-1 and survival of antiviral CD8(+) T cells during chronic infection. Immunity (2014) 41:802–14. 10.1016/j.immuni.2014.10.013 25464856 PMC4270830

[15] Man K Gabriel SS Liao Y Gloury R Preston S Henstridge DC. Transcription factor IRF4 promotes CD8(+) T cell exhaustion and limits the development of memory-like T cells during chronic infection. Immunity (2017) 47:1129–41 e1125. 29246443 10.1016/j.immuni.2017.11.021

[16] Pearce EL Mullen AC Martins GA Krawczyk CM Hutchins AS Zediak VP. Control of effector CD8+ T cell function by the transcription factor Eomesodermin. Science (2003) 302:1041–3. 10.1126/science.1090148 14605368

[17] Intlekofer AM Takemoto N Wherry EJ Longworth SA Northrup JT Palanivel VR. Effector and memory CD8+ T cell fate coupled by T-bet and eomesodermin. Nat Immunol. (2005) 6:1236–44. 10.1038/ni1268 16273099

[18] Buggert M Tauriainen J Yamamoto T Frederiksen J Ivarsson MA Michaelsson J. T-bet and Eomes are differentially linked to the exhausted phenotype of CD8+ T cells in HIV infection. PLoS Pathog (2014) 10:e1004251. 10.1371/journal.ppat.1004251 25032686 PMC4102564

[19] Li J Lee Y Li Y Jiang Y Lu H Zang W. Co-inhibitory molecule B7 superfamily member 1 expressed by tumor-infiltrating myeloid cells induces dysfunction of anti-tumor CD8(+) T Cells. Immunity (2018) 48:773–86 e775. 10.1016/j.immuni.2018.03.018 29625896

[20] Zhu Y Ju S Chen E Dai S Li C Morel P. T-bet and eomesodermin are required for T cell-mediated antitumor immune responses. J Immunol. (2010) 185:3174–83. 10.4049/jimmunol.1000749 20713880

[21] Sawada S Scarborough JD Killeen N Littman DR. A lineage-specific transcriptional silencer regulates CD4 gene expression during T lymphocyte development. Cell (1994) 77:917–29. 10.1016/0092-8674(94)90140-6 8004678

[22] Subramanian A Tamayo P Mootha VK Mukherjee S Ebert BL Gillette MA. Gene set enrichment analysis: a knowledge-based approach for interpreting genome-wide expression profiles. Proc Natl Acad Sci USA (2005) 102:15545–50. 10.1073/pnas.0506580102 16199517 PMC1239896

[23] Liu T Ortiz JA Taing L Meyer CA Lee B Zhang Y. Cistrome: an integrative platform for transcriptional regulation studies. Genome Biol (2011) 12:R83. 10.1186/gb-2011-12-8-r83 21859476 PMC3245621

[24] Wherry EJ Ha SJ Kaech SM Haining WN Sarkar S Kalia V. Molecular signature of CD8+ T cell exhaustion during chronic viral infection. Immunity (2007) 27:670–84. 10.1016/j.immuni.2007.09.006 17950003

[25] Jin HT Anderson AC Tan WG West EE Ha SJ Araki K. Cooperation of Tim-3 and PD-1 in CD8 T-cell exhaustion during chronic viral infection. Proc Natl Acad Sci USA (2010) 107:14733–8. 10.1073/pnas.1009731107 20679213 PMC2930455

[26] Fourcade J Sun Z Benallaoua M Guillaume P Luescher IF Sander C. Upregulation of Tim-3 and PD-1 expression is associated with tumor antigen-specific CD8+ T cell dysfunction in melanoma patients. J Exp Med. (2010) 207:2175–86. 10.1084/jem.20100637 20819923 PMC2947081

[27] Bengsch B Ohtani T Khan O Setty M Manne S O'Brien S. Epigenomic-guided mass cytometry profiling reveals disease-specific features of exhausted CD8 T cells. Immunity (2018) 48:1029–45 e1025. 10.1016/j.immuni.2018.04.026 29768164 PMC6010198

[28] Wu T Ji Y Moseman EA Xu HC Manglani M Kirby M. The TCF1-Bcl6 axis counteracts type I interferon to repress exhaustion and maintain T cell stemness. Sci Immunol. (2016) 1:eaai8593. 10.1126/sciimmunol.aai8593 28018990 PMC5179228

[29] Ejrnaes M Filippi CM Martinic MM Ling EM Togher LM Crotty S. Resolution of a chronic viral infection after interleukin-10 receptor blockade. J Exp Med. (2006) 203:2461–72. 10.1084/jem.20061462 17030951 PMC2118120

[30] Doering TA Crawford A Angelosanto JM Paley MA Ziegler CG Wherry EJ. Network analysis reveals centrally connected genes and pathways involved in CD8+ T cell exhaustion versus memory. Immunity (2012) 37:1130–44. 10.1016/j.immuni.2012.08.021 23159438 PMC3749234

[31] Wang S Sun H Ma J Zang C Wang C Wang J. Target analysis by integration of transcriptome and ChIP-seq data with BETA. Nat Protoc. (2013) 8:2502–15. 10.1038/nprot.2013.150 24263090 PMC4135175

[32] Iwata S Mikami Y Sun HW Brooks SR Jankovic D Hirahara K The Transcription Factor T-bet Limits Amplification of Type I IFN Transcriptome and Circuitry in T Helper 1 Cells. Immunity. (2017) 46:983–91 e984. 10.1016/j.immuni.2017.05.005 28623086 PMC5523825

[33] Minnie SA Kuns RD Gartlan KH Zhang P Wilkinson AN Samson L. Myeloma escape after stem cell transplantation is a consequence of T-cell exhaustion and is prevented by TIGIT blockade. Blood (2018) 132:1675–88. 10.1182/blood-2018-01-825240 30154111

[34] Li J Shayan G Avery L Jie HB Gildener-Leapman N Schmitt N. Tumor-infiltrating Tim-3(+) T cells proliferate avidly except when PD-1 is co-expressed: Evidence for intracellular cross talk. Oncoimmunology (2016) 5:e1200778. 10.1080/2162402X.2016.1200778 27853635 PMC5087305

[35] Sponaas AM Yang R Rustad EH Standal T Thoresen AS Dao Vo C. PD1 is expressed on exhausted T cells as well as virus specific memory CD8+ T cells in the bone marrow of myeloma patients. Oncotarget (2018) 9:32024–35. 10.18632/oncotarget.25882 30174794 PMC6112830

