# Supplementary material for: High Levels of Eomes Promote Exhaustion of Anti-tumor CD8+ T Cells
Source: Front Immunol. 2018 Dec 18;9:2981. doi: 10.3389/fimmu.2018.02981 (PMC6305494; doi:10.3389/fimmu.2018.02981)
Supplement: Supplementary file 4 [file Data_Sheet_1.PDF]

Figure S1.

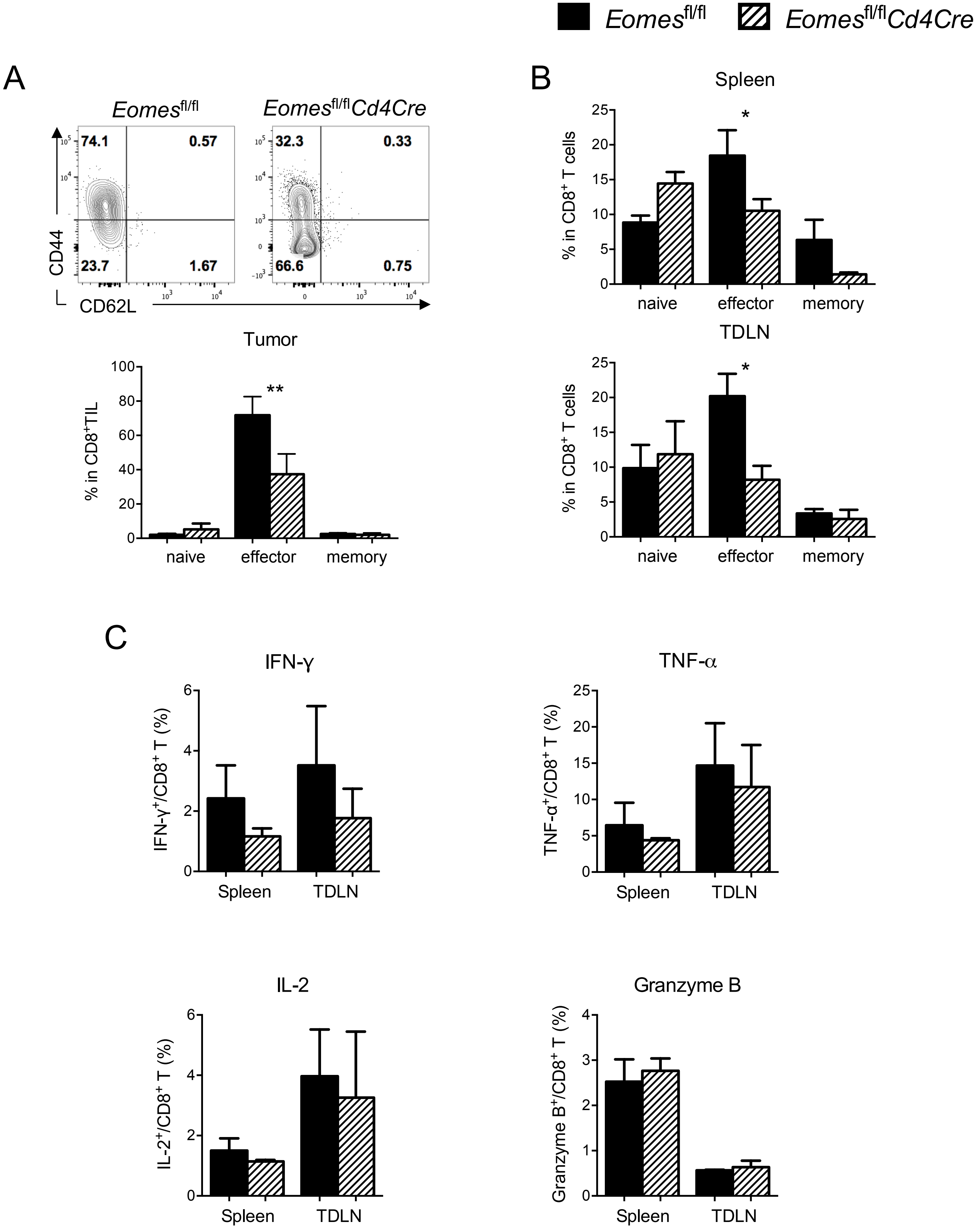

Eomesfl/fl
Eomesfl/flCd4Cre
A
Eomesfl/fl
Eomesfl/flCd4Cre
74.1
0.57
23.7
1.67
32.3
0.33
66.6
0.75
CD44
CD62L
Tumor
% in CD8+TIL
**
naive
effector
memory
B
Spleen
% in CD8+ T cells
*
naive
effector
memory
TDLN
% in CD8+ T cells
*
naive
effector
memory
C
IFN-γ
IFN-γ+/CD8+ T (%)
Spleen
TDLN
TNF-α
TNF-α+/CD8+ T (%)
Spleen
TDLN
IL-2
IL-2+/CD8+ T (%)
Spleen
TDLN
Granzyme B
Granzyme B+/CD8+ T (%)
Spleen
TDLN

Figure S1. Phenotypic analysis of $CD8^+$ T cells in spleen, TDLN and tumor of *Eomes*$^{fl/fl}$ vs. *Eomes*$^{fl/fl}$*Cd4Cre* mice. (A) Percentage of naïve ($CD62L^+CD44^-$), effector ($CD62L^-CD44^+$) and memory ($CD62L^+CD44^+$) cells in total $CD8^+$ T cells in the tumor of E.G7-bearing *Eomes*$^{fl/fl}$ vs. *Eomes*$^{fl/fl}$*Cd4Cre* mice on Day 21. (B) Percentage of naïve ($CD62L^+CD44^-$), effector ($CD62L^-CD44^+$) and memory ($CD62L^+CD44^+$) cells in total $CD8^+$ T cells in the spleen and tumor-draining lymph node (TDLN) of E.G7-bearing *Eomes*$^{fl/fl}$ vs. *Eomes*$^{fl/fl}$*Cd4Cre* mice on Day 21. (C) Production of IFN-γ, TNF-α, IL-2 and Granzyme B after PMA/ ionomycin stimulation by $CD8^+$ T cells in the spleen and TDLN of E.G7-bearing *Eomes*$^{fl/fl}$ vs. *Eomes*$^{fl/fl}$*Cd4Cre* mice on Day 21. Data are pooled from 2 independent experiments with 4-6 mice per group. Error bars denote mean ± SEM. Statistical analysis was performed using ordinary One-way ANOVA analysis followed by multiple comparisons. *$p<0.05$, **$p<0.01$.

Figure S2.

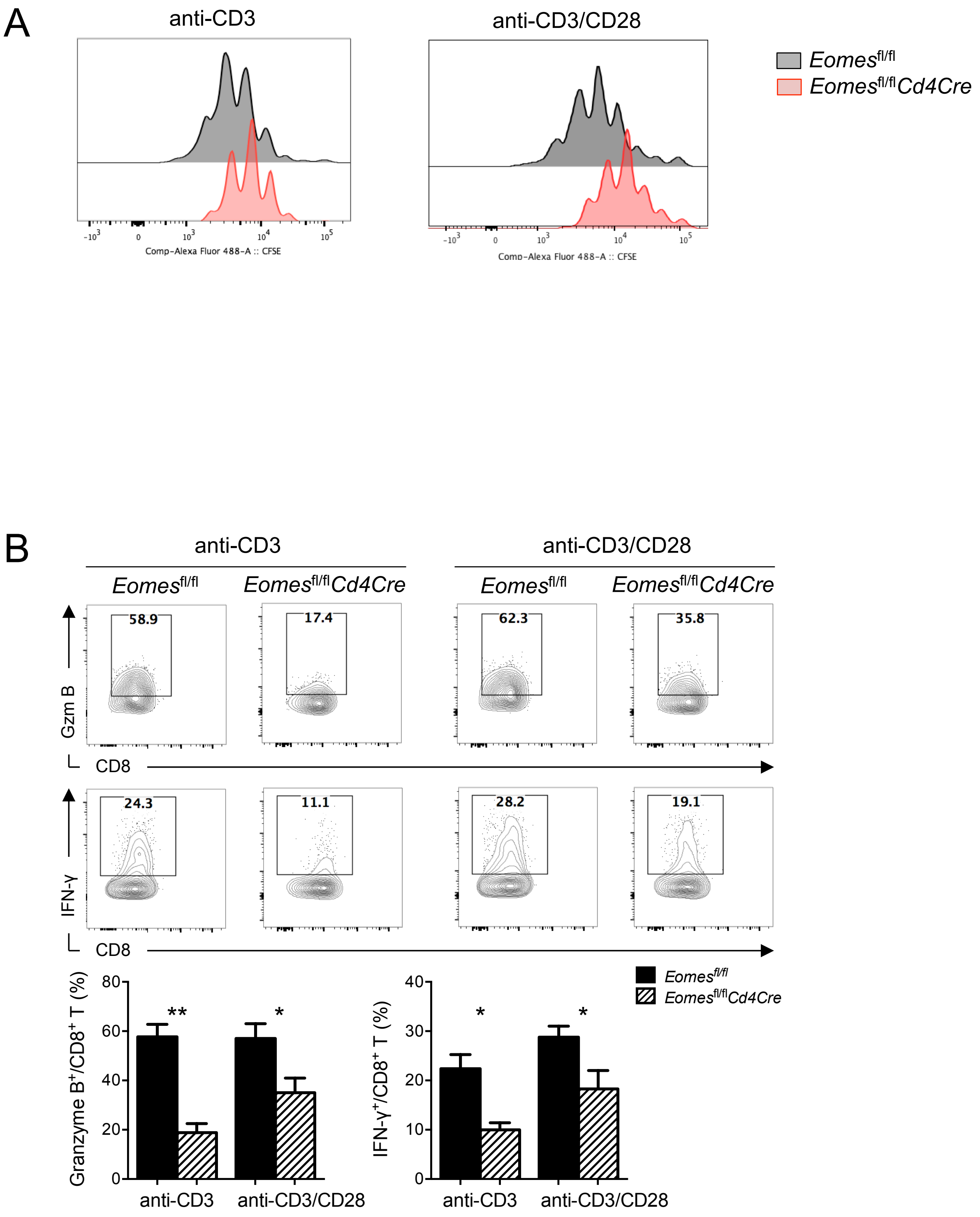
A
anti-CD3
anti-CD3/CD28
Comp-Alexa Fluor 488-A :: CFSE
Comp-Alexa Fluor 488-A :: CFSE
Eomes fl/fl
Eomes fl/fl Cd4Cre
B
anti-CD3
anti-CD3/CD28
Eomes fl/fl
Eomes fl/fl Cd4Cre
Eomes fl/fl
Eomes fl/fl Cd4Cre
58.9
17.4
62.3
35.8
Gzm B
CD8
24.3
11.1
28.2
19.1
IFN-γ
CD8
Granzyme B+/CD8+ T (%)
**
*
anti-CD3
anti-CD3/CD28
IFN-γ+/CD8+ T (%)
*
*
anti-CD3
anti-CD3/CD28
Eomes fl/fl
Eomes fl/fl Cd4Cre

Figure S2. *In vitro* stimulation of CD8$^+$ T cells isolated from *Eomes*$^{fl/fl}$ or *Eomes*$^{fl/fl}$*Cd4Cre* mice. CD8$^+$ T cells were isolated from spleen and lymph nodes of *Eomes*$^{fl/fl}$ or *Eomes*$^{fl/fl}$*Cd4Cre* mice, labeled with CFSE and cultured in 96-well plate coated with 1μg/mL anti-CD3 or 1μg/mL anti-CD3+1μg/mL anti-CD28 (10^5 per well) for 3 days. To detect cytokine production, Golgi Plug was added 4 hours prior to harvest and cytokine production were measured by intracellular flow cytometric analysis. (A) Proliferation of CD8$^+$ T cells isolated from *Eomes*$^{fl/fl}$ and *Eomes*$^{fl/fl}$*Cd4Cre* mice marked by CFSE dilution. (B) Production of Granzyme B and IFN-γ by CD8$^+$ T cells isolated from *Eomes*$^{fl/fl}$ and *Eomes*$^{fl/fl}$*Cd4Cre* mice. Data are pooled from 2 independent experiments. Error bars denote mean ± SEM. Statistical analysis was performed using ordinary One-way ANOVA analysis followed by multiple comparisons. *$p<0.05$, **$p<0.01$.

Figure S3.

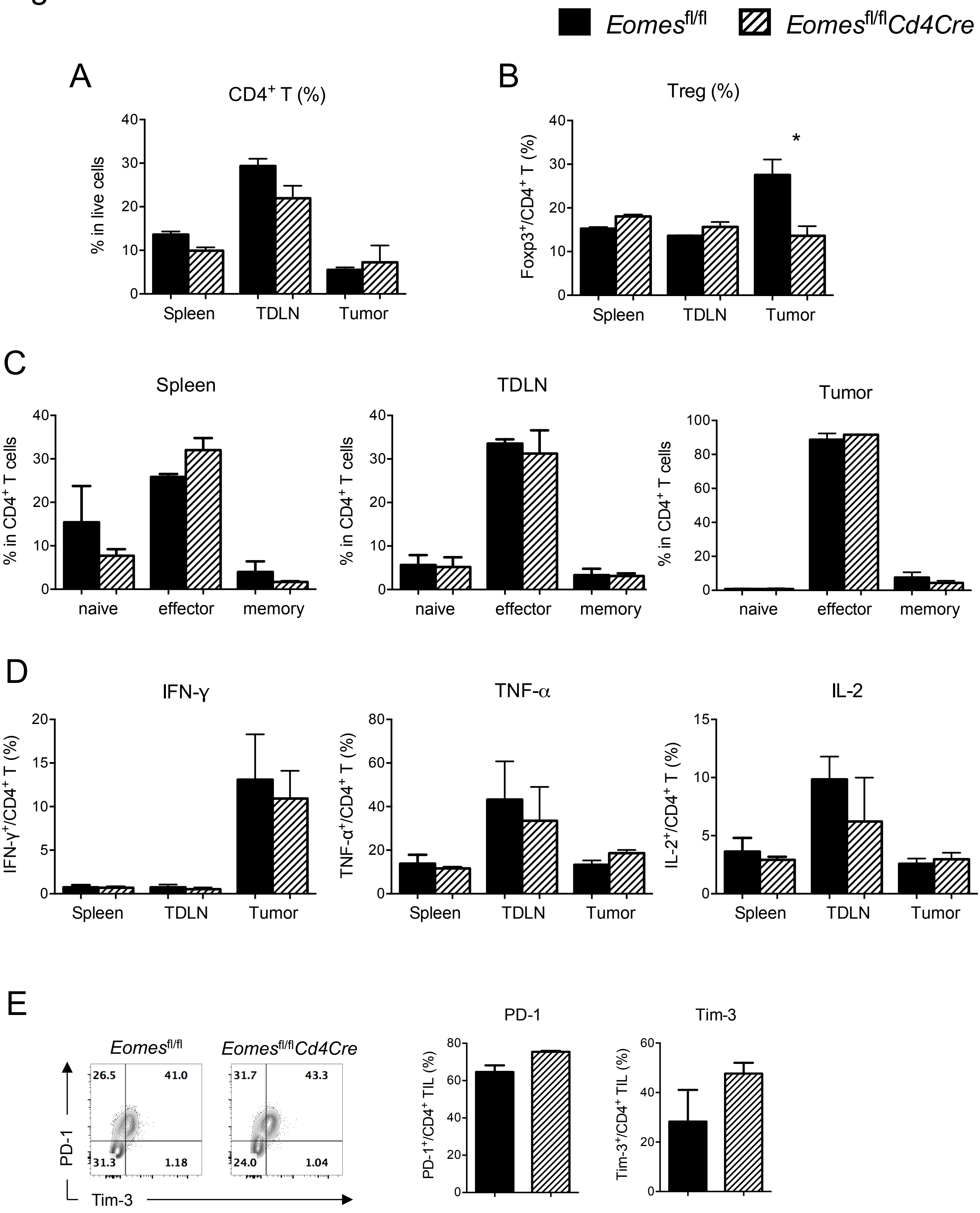

Eomes^fl/fl
Eomes^fl/fl Cd4Cre
A
CD4+ T (%)
% in live cells
Spleen
TDLN
Tumor
B
Treg (%)
Foxp3+/CD4+ T (%)
*
C
Spleen
TDLN
Tumor
% in CD4+ T cells
naive
effector
memory
D
IFN-γ
TNF-α
IL-2
IFN-γ+/CD4+ T (%)
TNF-α+/CD4+ T (%)
IL-2+/CD4+ T (%)
E
Eomes^fl/fl
Eomes^fl/fl Cd4Cre
26.5
41.0
31.3
1.18
31.7
43.3
24.0
1.04
PD-1
Tim-3
PD-1+/CD4+ TIL (%)
Tim-3+/CD4+ TIL (%)

Figure S3. Phenotypic analysis of $CD4^+$ T cells in spleen, TDLN and tumor of *Eomes*$^{fl/fl}$ vs. *Eomes*$^{fl/fl}$*Cd4Cre* mice. (A) Frequency of $CD4^+$ T cells in total live cells isolated from spleen, TDLN and tumor of *Eomes*$^{fl/fl}$ vs. *Eomes*$^{fl/fl}$*Cd4Cre* mice on Day 21. (B) Percentage of regulatory T cells ($Foxp3^+$) in $CD4^+$ T cells from spleen, TDLN and tumor of *Eomes*$^{fl/fl}$ vs. *Eomes*$^{fl/fl}$*Cd4Cre* mice on Day 21. (C) Percentage of naïve ($CD62L^+CD44^-$), effector ($CD62L^-CD44^+$) and memory ($CD62L^+CD44^+$) cells in total $CD4^+$ T cells in the spleem, TDLN and tumor of *Eomes*$^{fl/fl}$ vs. *Eomes*$^{fl/fl}$*Cd4Cre* mice on Day 21. (D) Production of IFN-γ, TNF-α and IL-2 after PMA/ionomycin stimulation by $CD4^+$ T cells in the spleen, TDLN and tumor of *Eomes*$^{fl/fl}$ vs. *Eomes*$^{fl/fl}$*Cd4Cre* mice on Day 21. (E) Expression of PD-1 and Tim-3 on $CD4^+$ T cells in the tumor of *Eomes*$^{fl/fl}$ vs. *Eomes*$^{fl/fl}$*Cd4Cre* mice on Day 21. Data are pooled from 2 independent experiments with 4-6 mice per group. Error bars denote mean ± SEM. Statistical analysis was performed using ordinary One-way ANOVA analysis followed by multiple comparisons. $^*p<0.05$.

Figure S4.

A

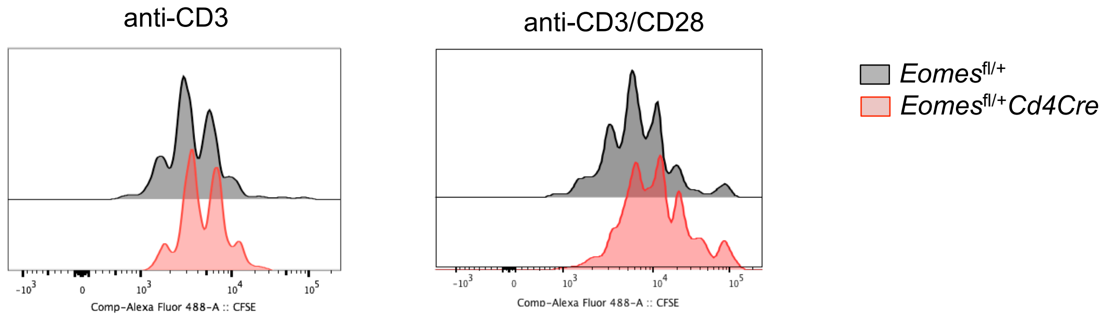

anti-CD3
anti-CD3/CD28
Eomes fl/+
Eomes fl/+ Cd4Cre
Comp-Alexa Fluor 488-A :: CFSE

B

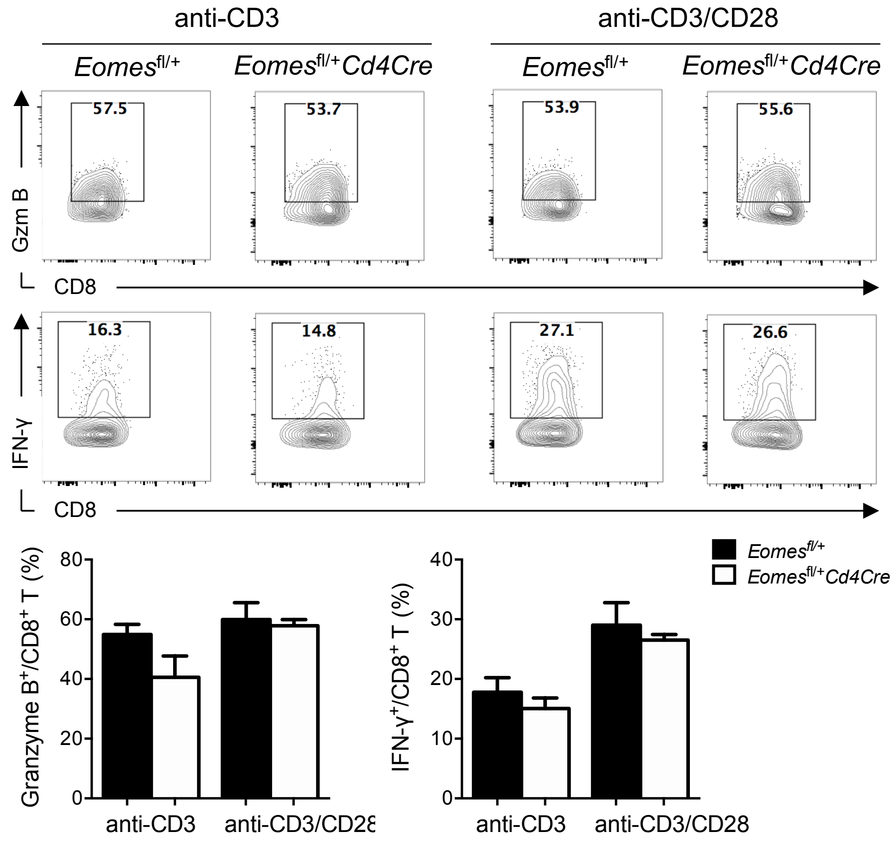

anti-CD3
anti-CD3/CD28
Eomes fl/+
Eomes fl/+ Cd4Cre
57.5
53.7
53.9
55.6
Gzm B
CD8
16.3
14.8
27.1
26.6
IFN-γ
CD8
Granzyme B+/CD8+ T (%)
IFN-γ+/CD8+ T (%)
anti-CD3
anti-CD3/CD28

Figure S4. *In vitro* stimulation of CD8$^+$ T cells isolated from *Eomes*$^{fl/+}$ or *Eomes*$^{fl/+}$*Cd4Cre* mice. CD8$^+$ T cells were isolated from spleen and lymph nodes of *Eomes*$^{fl/+}$ or *Eomes*$^{fl/+}$*Cd4Cre* mice, labeled with CFSE and cultured in 96-well plate coated with 1μg/mL anti-CD3 or 1μg/mL anti-CD3+1μg/mL anti-CD28 (10^5 per well) for 3 days. To detect cytokine production, Golgi Plug was added 4 hours prior to harvest and cytokine production were measured by intracellular flow cytometric analysis. (A) Proliferation of CD8$^+$ T cells isolated from *Eomes*$^{fl/+}$ and *Eomes*$^{fl/+}$*Cd4Cre* mice marked by CFSE dilution. (B) Production of Granzyme B and IFN-γ by CD8$^+$ T cells isolated from *Eomes*$^{fl/+}$ and *Eomes*$^{fl/+}$*Cd4Cre* mice. Data are pooled from 2 independent experiments. Error bars denote mean ± SEM. Statistical analysis was performed using ordinary One-way ANOVA analysis followed by multiple comparisons. *$p<0.05$, **$p<0.01$.

Figure S5.

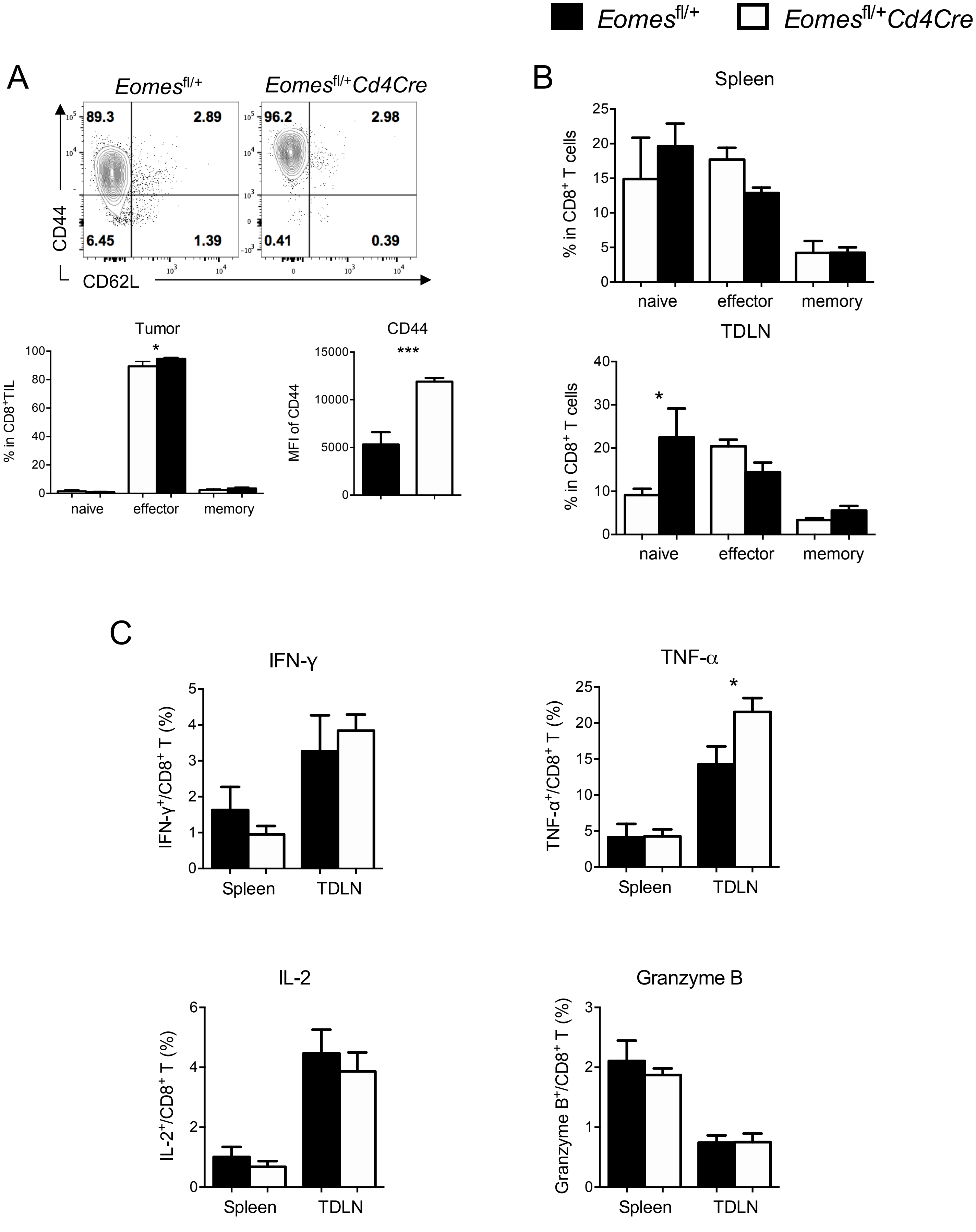

Eomes^fl/+
Eomes^fl/+Cd4Cre
A
Eomes^fl/+
Eomes^fl/+Cd4Cre
89.3
2.89
6.45
1.39
96.2
2.98
0.41
0.39
CD44
CD62L
Tumor
% in CD8+TIL
naive
effector
memory
CD44
MFI of CD44
B
Spleen
% in CD8+ T cells
naive
effector
memory
TDLN
% in CD8+ T cells
naive
effector
memory
C
IFN-γ
IFN-γ+/CD8+ T (%)
Spleen
TDLN
TNF-α
TNF-α+/CD8+ T (%)
Spleen
TDLN
IL-2
IL-2+/CD8+ T (%)
Spleen
TDLN
Granzyme B
Granzyme B+/CD8+ T (%)
Spleen
TDLN

Figure S5. Phenotypic analysis of $CD8^+$ T cells in spleen, TDLN and tumor of *Eomes*$^{fl/+}$ vs. *Eomes*$^{fl/+}$*Cd4Cre* mice. (A) Percentage of naïve ($CD62L^+CD44^-$), effector ($CD62L^-CD44^+$) and memory ($CD62L^+CD44^+$) cells in total $CD8^+$ T cells together with CD44 expression on $CD8^+$ T cells in the tumor of E.G7-bearing *Eomes*$^{fl/+}$ vs. *Eomes*$^{fl/+}$*Cd4Cre* mice on Day 21. (B) Percentage of naïve ($CD62L^+CD44^-$), effector ($CD62L^-CD44^+$) and memory ($CD62L^+CD44^+$) cells in total $CD8^+$ T cells in the spleen and tumor-draining lymph node (TDLN) of E.G7-bearing *Eomes*$^{fl/+}$ vs. *Eomes*$^{fl/+}$*Cd4Cre* mice on Day 21. (C) Production of IFN-γ, TNF-α, IL-2 and Granzyme B after PMA/ionomycin stimulation by $CD8^+$ T cells in the spleen and TDLN of E.G7-bearing *Eomes*$^{fl/+}$ vs. *Eomes*$^{fl/+}$*Cd4Cre* mice on Day 21. Data are pooled from 2 independent experiments with 5-8 mice per group. Error bars denote mean ± SEM. Statistical analysis was performed using ordinary One-way ANOVA analysis followed by multiple comparisons. *$p<0.05$, ***$p<0.001$.

Figure S6.

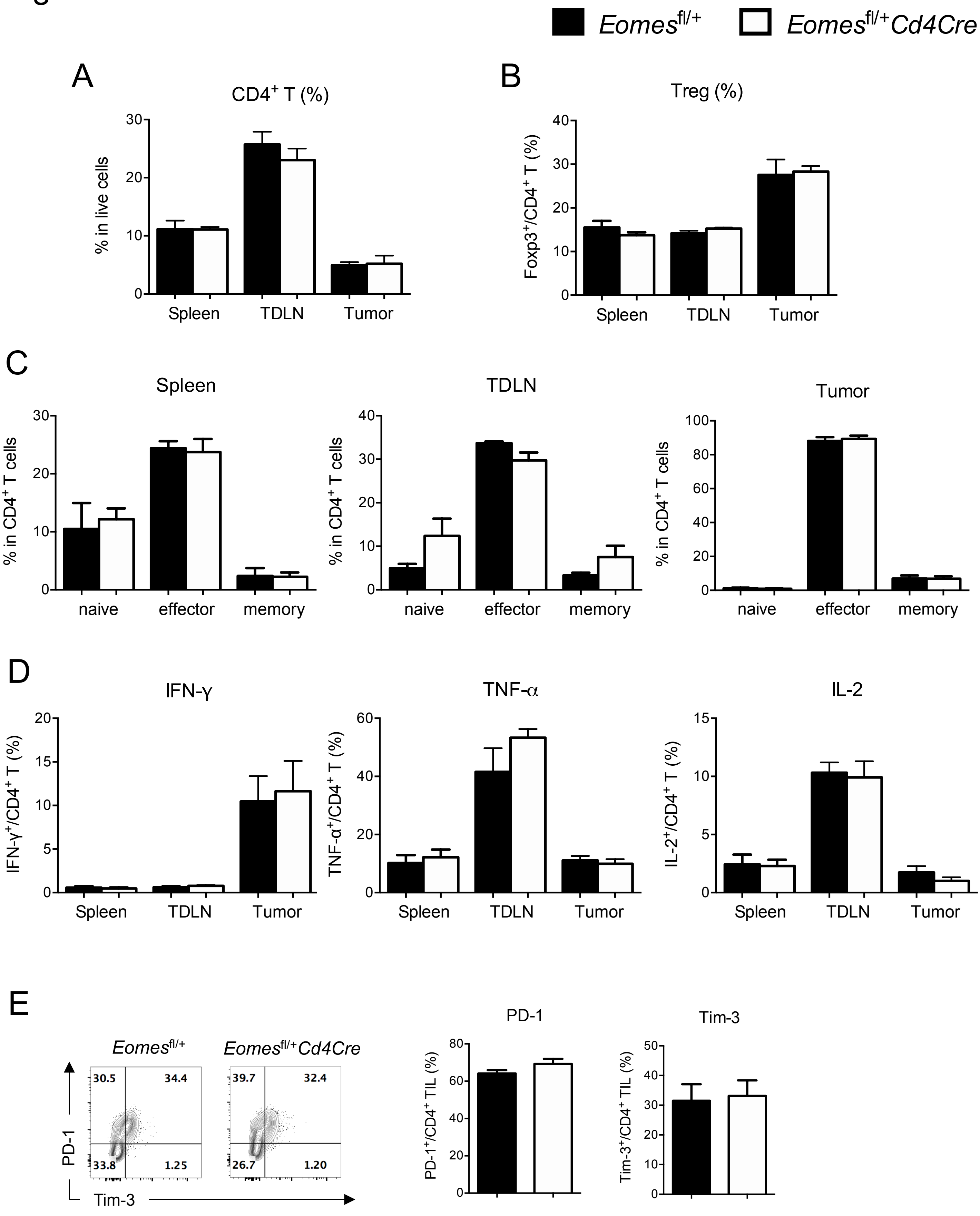

Eomesfl/+
Eomesfl/+Cd4Cre
A
CD4+ T (%)
% in live cells
0
10
20
30
Spleen
TDLN
Tumor
B
Treg (%)
Foxp3+/CD4+ T (%)
0
10
20
30
40
Spleen
TDLN
Tumor
C
Spleen
TDLN
Tumor
% in CD4+ T cells
0
10
20
30
40
60
80
100
naive
effector
memory
D
IFN-γ
TNF-α
IL-2
IFN-γ+/CD4+ T (%)
TNF-α+/CD4+ T (%)
IL-2+/CD4+ T (%)
0
5
10
15
20
40
60
Spleen
TDLN
Tumor
E
Eomesfl/+
Eomesfl/+Cd4Cre
30.5
34.4
33.8
1.25
39.7
32.4
26.7
1.20
PD-1
Tim-3
PD-1
Tim-3
PD-1+/CD4+ TIL (%)
Tim-3+/CD4+ TIL (%)
0
10
20
30
40
50
60
80

Figure S6. Phenotypic analysis of $CD4^+$ T cells in spleen, TDLN and tumor of *Eomes*$^{fl/+}$ vs. *Eomes*$^{fl/+}$*Cd4Cre* mice. (A) Frequency of $CD4^+$ T cells in total live cells isolated from spleen, TDLN and tumor of *Eomes*$^{fl/+}$ vs. *Eomes*$^{fl/+}$*Cd4Cre* mice on Day 21. (B) Percentage of regulatory T cells ($Foxp3^+$) in $CD4^+$ T cells from spleen, TDLN and tumor of *Eomes*$^{fl/+}$ vs. *Eomes*$^{fl/+}$*Cd4Cre* mice on Day 21. (C) Percentage of naïve ($CD62L^+CD44^-$), effector ($CD62L^-CD44^+$) and memory ($CD62L^+CD44^+$) cells in total $CD4^+$ T cells in the spleem, TDLN and tumor of *Eomes*$^{fl/+}$ vs. *Eomes*$^{fl/+}$*Cd4Cre* mice on Day 21. (D) Production of IFN-γ, TNF-α and IL-2 after PMA/ionomycin stimulation by $CD4^+$ T cells in the spleen, TDLN and tumor of *Eomes*$^{fl/+}$ vs. *Eomes*$^{fl/+}$*Cd4Cre* mice on Day 21. (E) Expression of PD-1 and Tim-3 on $CD4^+$ T cells in the tumor of *Eomes*$^{fl/+}$ vs. *Eomes*$^{fl/+}$*Cd4Cre* mice on Day 21. Data are pooled from 2 independent experiments with 5-8 mice per group. Error bars denote mean ± SEM. Statistical analysis was performed using ordinary One-way ANOVA analysis followed by multiple comparisons.

Figure S7.

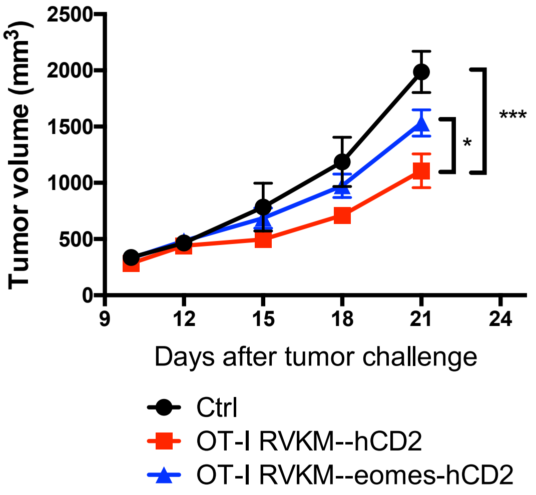

Tumor volume (mm3)
2500
2000
1500
1000
500
0
9
12
15
18
21
24
Days after tumor challenge
***
*
Ctrl
OT-I RVKM--hCD2
OT-I RVKM--eomes-hCD2

Figure S7. Eomes-overexpressing OT-I cells displayed impaired *in vivo* cytotoxicity. Mean tumor volume of E.G7-bearing mice (n=6 per group) adoptively transferred with 0.5×10^6 control OT-I or Eomes-overexpressing OT-I on Day 12. Ctrl (black), E.G7-bearing mice without OT-I transfer. OT-I RVKM--hCD2 (red), E.G7-bearing mice transferred with OT-I cells transfected with the empty vector. OT-I RVKM--eomes-hCD2 (blue), E.G7-bearing mice transferred with Eomes-overexpressing OT-I cells. Data are pooled from 2 independent experiments. Error bars denote mean ± SEM. Statistical analysis was performed using linear regression. *p<0.05, ***p<0.001.
